# Host plants influence the composition of the gut bacteria in *Henosepilachna vigintioctopunctata*

**DOI:** 10.1371/journal.pone.0224213

**Published:** 2019-10-18

**Authors:** Jing Lü, Wei Guo, Shimin Chen, Mujuan Guo, Baoli Qiu, Chunxiao Yang, Tengxiang Lian, Huipeng Pan

**Affiliations:** 1 Key Laboratory of Bio-Pesticide Innovation and Application of Guangdong Province, South China Agricultural University, Guangzhou, China; 2 State Key Laboratory for Conservation and Utilization of Subtropical Agro-bioresources, South China Agricultural University, Guangzhou, China; Free University of Bozen-Bolzano, ITALY

## Abstract

The gut bacteria of insects positively influence the physiology of their host, however, the dynamics of this complicated ecosystem are not fully clear. To improve our understanding, we characterized the gut prokaryotic of *Henosepilachna vigintioctopunctata* that fed on two host plants, *Solanum melongena* (referred to as QZ hereafter) and *Solanum nigrum* (referred to as LK hereafter), by sequencing the V3-V4 hypervariable region of the 16S rRNA gene using the Illumina MiSeq system. The results revealed that the gut bacterial composition varied between specimens that fed on different host plants. The unweighted pair group method with arithmetic mean analyses and principal coordinate analysis showed that the bacterial communities of the LK and QZ groups were distinct. Four phyla (Proteobacteria, Bacteroidetes, Firmicutes, and Actinobacteria) were present in all *H*. *vigintioctopunctata* gut samples. It is noteworthy that bacteria of the phylum Cyanobacteria were only found in the LK group, with a low relative abundance. Proteobacteria and *Enterobacteriaceae* were the predominant phylum and family, respectively, in both the LK and QZ groups. Linear discriminant analysis effect size (LEfSe) analyses showed that the QZ group enriched the Bacilli class and *Lactococcus* genus; while the LK group enriched the Alphaproteobacteria class and *Ochrobactrum* genus. PICRUSt analysis showed that genes predicted to be involved in xenobiotic biodegradation and metabolism, metabolism of other amino acids, signaling molecules, and interaction were significantly higher in the QZ group. Genes predicted to be involved in the metabolism of cofactors and vitamins were significantly higher in the LK group. Furthermore, the complexity of the network structure and the modularity were higher in the LK group than in the QZ group. This is the first study to characterize the gut bacteria of *H*. *vigintioctopunctat*, our results demonstrate that the two host plants tested had a considerable impact on bacterial composition in the gut of *H*. *vigintioctopunctata* and that the bacterial communities were dominated by relatively few taxa.

## Introduction

Insects harbor indigenous bacterial communities in their gut. Recently, the microbial communities associated with insect guts have been garnering interest, largely because of their ecological and economic importance. Microbes can play important roles in a myriad of host functions, including development [1], food digestion and energy extraction [2,3], defense against natural enemies [4], immune responses [5], insecticide resistance [6], production of essential vitamins, and gut physiology [4]. For example, microorganisms possess metabolic characteristics that are often absent in insects; thus, they can help the insects adapt to host plants [7]. This is especially obvious in herbivorous insects, due to the wide range of secondary materials present in plant tissues. Hence, insects have evolved a battery of strategies to surmount plant defenses [8].

Over a long period of coevolution, a symbiotic interplay has formed between insects and their gut bacteria. Insect gut bacteria have demonstrated some plasticity, possessing the ability to quickly adapt to changes in the insect diet, or to changes in their population structure [9–11]. This adaptive capacity can help insects by enabling them to exploit different kinds of food resources and laying the foundation for the development of host-associated differentiation. Thus, a complete description of the bacterial communities of the gut is pivotal for an integrated understanding of the ecology and biology of insect hosts, and could possibly result in the research and development of new pest management strategies.

*Henosepilachna vigintioctopunctata* (Fabricius) (Coleoptera: Coccinellidae), is an important pest in Asia [12]. In China, *H*. *vigintioctopunctata* is spread across the entire Country [13]. In recent years, host plants of *H*. *vigintioctopunctata* are being grown year-round, facilitated by global warming, the development of trade, and the expansion of the cultivated area of protected vegetables. This has increased the damage caused by this pest. *H*. *vigintioctopunctata* colonizes many different species of plants including eggplant *Solanum melongena*, tomato *Solanum lycopersicum*, potato *Solanum tuberosum*, pepper *Capsicum annuum*, cucumber *Cucumis sativus*, waxgourd *Benincasa hispida*, black nightshade *Solanum nigrum*, winter cherry *Physalis alkekengi*, and tobacco *Nicotania tabacum* [13]. One recent study has shown that the fecundity of *H*. *vigintioctopunctata* adults that fed on *S*. *nigrum* was remarkably higher than that fed on three *S*. *melongena* cultivars. The finite rate of increase, intrinsic rate of increase, and net reproductive rate of *H*. *vigintioctopunctata* were significantly higher when reared on *S*. *nigrum* than comparable values when reared on two *S*. *melongena* cultivars, but did not differ from values when *H*. *vigintioctopunctata* were reared on another *S*. *melongena* cultivar [14]. Thus, to some extent, *S*. *nigrum* is a better host for *H*. *vigintioctopunctata* than *S*. *melongena*.

In recent years, culture-independent PCR amplification of 16S rRNA has become a reliable method for investigating the composition and abundance of gut bacteria [15]. The characterization of insect bacterial communities, in conjunction with information on host-associated variation in bacteria composition, is indispensable for an overall understanding of insect ecology, as well as for the development of new pest management strategies. The present study was implemented to ascertain the composition and diversity of the bacterial communities in the fourth instar *H*. *vigintioctopunctata* gut. In order to know more about the bacterial communities associated with *H*. *vigintioctopunctata*, this study also examined the changes in gut bacteria that two host plant species, *S*. *nigrum* and *S*. *melongena*, undergo as a result of this association.

## Materials and methods

### Insect rearing and sampling

Adults *H*. *vigintioctopunctata* were collected from *S*. *nigrum* at South China Agricultural University, Guangzhou, Guangdong Province (113°36′N, 23°17′E) in April 2018, and then reared under controlled conditions; temperature 25 ± 0.5°C, 80% relative humidity, and 14L:10D photoperiod [16]. The *H*. *vigintioctopunctata* colony was divided into two groups. One group was fed with *S*. *nigrum* leaves (LK group), and the other group was fed with *S*. *melongena* leaves (QZ group). After they fed on each host plant species for four generations, the guts of the fourth instar larvae were collected, respectively. *S*. *nigrum* and *S*. *melongena* (cv. Wanshengyuanshuai F1) were cultivated in a potting mix in 1.0 L pots (one plant/pot) under natural light and controlled temperature (22–28°C) in a glasshouse. The leaves were collected, rinsed with ddH_2_O, and then dried with filter paper before feeding them to *H*. *vigintioctopunctata* during the whole experimental period. Sufficient leaves were provided for each individual.

*H*. *vigintioctopunctata* guts were collected from the fourth instar larvae of both the LK and QZ groups, regardless of sex. Specifically, the guts from 30 fourth instar individuals were dissected as one replicate and three replicates were used for each group. The fourth instar larvae surface was disinfected with 75% ethanol for 90 s and rinsed with ddH_2_O. Following dissection, the guts were collected in a 1.5 mL centrifuge tube and then frozen at -80°C prior to DNA extraction.

### DNA extraction, amplicon generation, and library preparation

Genomic DNA was isolated from the guts (the guts dissected from 30 fourth instar individuals as one replicate) using the HiPure Soil DNA Mini Kit (Magen, Guangzhou, China), according to the manufacturer’s instructions. The prokaryotic 16S rRNA V3-V4 hypervariable regions were amplified from a total of 20–30 ng of metagenomic DNA and were sequenced with the forward primer 5′-CCTACGGRRBGCASCAGKVRVGAAT-3′ and reverse primer 5′-GGACTACNVGGGTWTCTAATCC-3′ [17]. Meanwhile, indexed adapters were added to the ends of the 16S rRNA amplicons to generate indexed libraries ready for downstream next-generation sequencing on an Illumina MiSeq system [18].

### Illumina MiSeq sequencing

DNA library concentrations were validated using a Qubit 3.0 Fluorometer. The library was quantified and adjusted to 10 nM. DNA libraries were multiplexed and loaded on an Illumina MiSeq instrument according to manufacturer’s instructions (Illumina, San Diego, CA, USA). Sequencing was performed using PE250/300 paired-end; image analysis and base calling were conducted with the MiSeq control software embedded in the MiSeq instrument. The overlapped full V3-V4 tags generated from PE reads for each line described above have been deposited at the NCBI database under accession number PRJNA503516.

### Data analysis

QIIME (Version 1.9.0) was used for the raw sequence data analysis. Briefly, low quality sequences with sequence length < 200 bp, and mean quality score ≥ 20, were removed. Then, the chimeric sequences were removed using the UCHIME algorithm. The effective sequences were clustered into operational taxonomic units (OTUs) using VSEARCH (Version 1.9.6) against the Silva 132 database, based on 97% sequence similarity. The Shannon, Ace, and Chao1 indices were calculated in QIIME and used to compare gut bacterial alpha diversity. Weighted and unweighted UniFrac and principal coordinate analysis were calculated for describing the beta diversity.

The Adonis test was performed in R 3.5.1 with the vegan package to analyze differences in the entire bacterial communities of the LK and QZ samples. STAMP V2.1.3 was used to analyze the differences between LK and QZ samples at the genus level.

Linear discriminant analysis (LDA) effect size (LEfSe) was performed to find significantly abundant bacterial taxa within these two groups. The factorial Kruskal-Wallis sum-rank test (*α* = 0.05) was used to identify characterization of the features of the bacterial communities with significant differential abundance between categories, and then LDA was performed to estimate the effect size of each feature [19].

Functional prediction of intestinal bacterial composition was carried out based on the reference sequence library of 16S rRNA gene. The PICRUSt algorithm was used to infer the functions of the bacterial communities through the KEGG (Kyoto Encyclopedia of Genes and Genomes) database [20].

Association network analyses were performed to understand the relationship among the genera using R software and Gephi. Spearman’s correlation coefficient was greater than 0.6, while a *p*-value of less than 0.05 was considered to be a valid interaction network. The network topological properties are calculated using Gephi [21].

### Statistical analysis

The means of two independent groups were compared by Student’s *t-*test, using SPSS 17 (SPSS Inc., Chicago, IL, USA). Results were considered statistically significant when *p* < 0.05.

## Results

### Overall structural changes in the bacterial communities

The number of valid reads varied among different samples (Table 1). In total, 298,591 high-quality reads with an average length of 463 bp were obtained. Rarefaction analysis indicated that the number of species increased rapidly before reaching a plateau (S1 Fig). Bacterial diversity was measured based on OTUs; four parameters demonstrated that there was no diversity difference between the LK and QZ groups, although the Ace and Chao1 index suggested that the bacterial diversity of the LK group was higher than that of the QZ group (Table 1). Principal coordinate and unweighted pair group method with arithmetic mean analyses indicated that the entire bacterial communities of the LK and QZ samples were clearly distinct from each other (Adonis test, *p* < 0.05; S2 and S3 Figs), indicating that the host plant had a significant impact on the fourth instar *H*. *vigintioctopunctata* gut bacterial communities.

**Table 1:** Alpha diversity of the gut bacterial community in the *Solanum melongena* (QZ) and *Solanum nigrum* (LK) groups.

Sample	Number of valid reads	Ace	Chao	Shannon	Simpson
LK	46388 ± 3747	73.26 ± 2.45	72.90 ± 3.15	2.61 ± 0.16	0.69 ± 0.02
QZ	53142 ± 5927	65.28 ± 0.21	64.38 ± 0.78	2.78 ± 0.09	0.73 ± 0.01
*P*	0.390	0.082	0.058	0.395	0.175
*F*	0.470	9.321	5.381	0.581	0.336

### Bacterial composition

**Fig 1:**
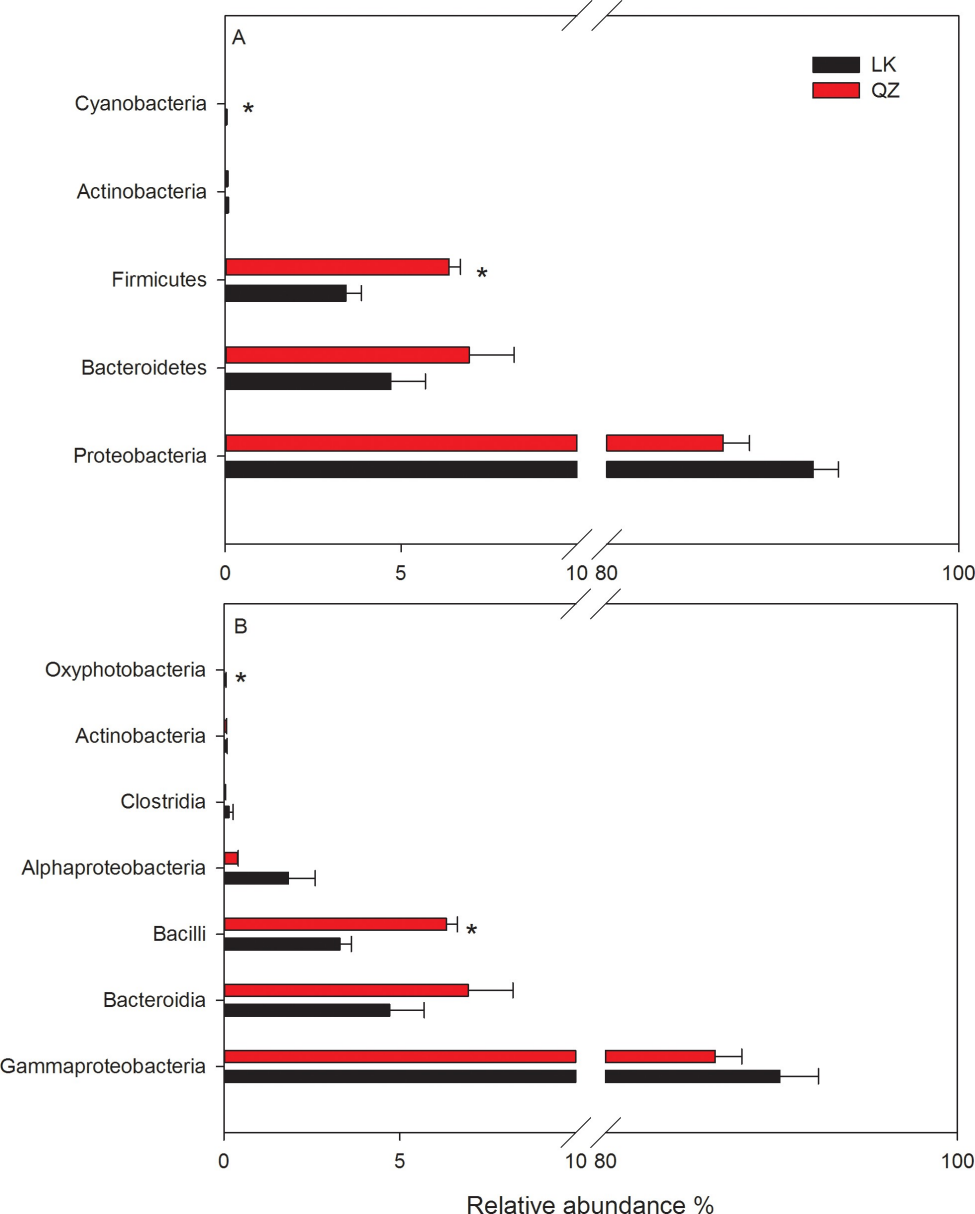
Relative abundance (%) of gut bacterial (A) phyla and (B) classes in samples from the *Solanum melongena* (QZ) and *Solanum nigrum* (LK) groups.

Four phyla (Proteobacteria, Bacteroidetes, Firmicutes, and Actinobacteria) were present in all the samples. Proteobacteria, the most abundant phylum, accounted for 91.74 ± 1.43% and 86.63 ± 1.49% of the total bacteria in the LK and QZ groups, respectively (Fig 1A, S1 Table). In addition, the relative abundance of Firmicutes was significantly higher in the QZ group than that in the LK group (Fig 1A, S1 Table), while the relative abundance of Actinobacteria was < 1.0% in both the LK and QZ groups, with no difference between the two. Interestingly, the phylum Cyanobacteria was only found in the LK group (0.04 ± 0.01%), although with low relative abundance (Fig 1A, S1 Table).

Six classes (Gammaproteobacteria, Bacteroidia, Bacilli, Alphaproteobacteria, Clostridia, and Actinobacteria) were present in all *H*. *vigintioctopunctata* gut samples. The most dominant class in the *H*. *vigintioctopunctata* gut was Gammaproteobacteria, which accounted for 89.91 ± 2.19% and 86.24 ± 1.51% in the LK and QZ groups, respectively (Fig 1B, S2 Table). Other dominant classes were Bacteroidia and Bacilli, with the relative abundance of Bacilli significantly higher in the QZ group than in the LK group (Fig 1B, S2 Table). In addition, the average percentage of Alphaproteobacteria in the LK and QZ groups was 1.83 ± 0.76% and 0.39 ± 0.02%, respectively. The average percentage of Clostridia and Actinobacteria was low (< 0.15%) in both the LK and QZ groups (Fig 1B, S2 Table). The class Oxyphotobacteria was only found in the LK group (Fig 1B, S2 Table).

**Fig 2:**
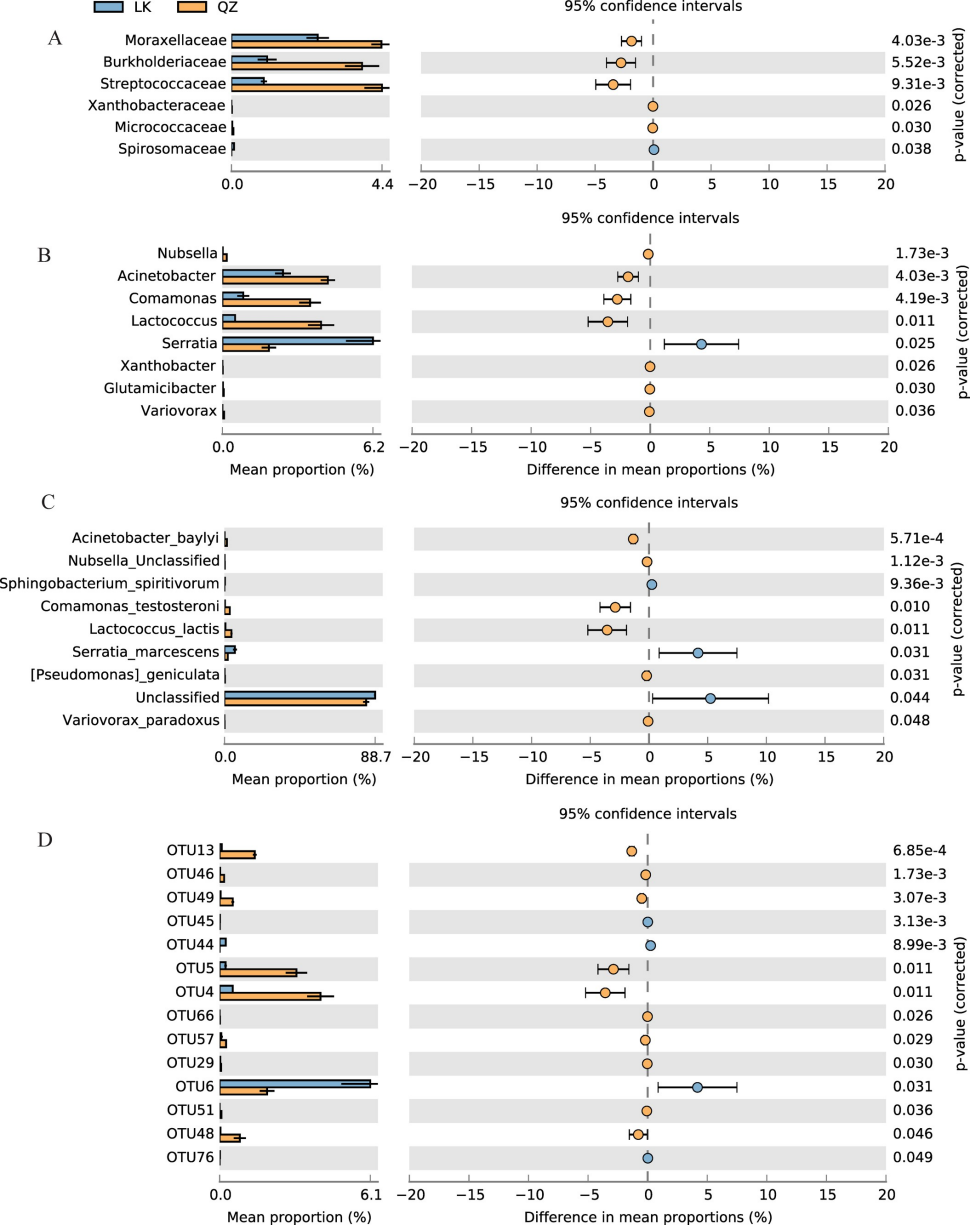
The relative abundance of gut bacteria at the class (A), genus (B), species (C), and OTU (D) level in the *Solanum melongena* (QZ) and *Solanum nigrum* (LK) groups. The colored circles, corresponding to the right column (yellow and blue), repr-esent 95% confidence intervals calculated using Welch's t-test. The error bars signify the calculated standard variation of triplicate samples.

The number and relative abundance of bacterial families varied in the LK and QZ groups, with an average number of families of 30.33 ± 0.88 and 28.00 ± 0.58 in the LK and QZ groups, respectively (Table 2). *Enterobacteriaceae* was the predominant bacterial family in the LK and QZ groups, followed by the family *Pseudomonadaceae* (S4 Fig, S3 Table). The relative abundance of *Moraxellaceae*, *Burkholderiaceae*, *Streptococcaceae*, *Xanthobacteraceae*, and *Micrococcaceae* was much higher in the QZ group than in the LK group, whereas the relative abundance of *Spirosomaceae* was much higher in the LK group than in the QZ group (Fig 2A, S3 Table).

**Table 2:** Number of different gut bacteria taxonomic categories in the *Solanum melongena* (QZ) and *Solanum nigrum* (LK) groups.

Sample	Phylum	Class	Order	Family	Genus	Species	OTUs
LK	5 ± 0	7 ± 0	18.67 ± 0.33a	30.33 ± 0.88	42.33+3.18	25.33 ± 0.88a	71.00 ± 2.89a
QZ	4 ± 0	6 ± 0	16.00 ± 0.58b	28.00 ± 0.58	38.00+1.15	21.00 ± 0.58b	61.33 ± 0.33b
*P*			0.016	0.091	0.269	0.015	0.029
*F*			0.400	0.442	5.083	0.727	2.991

Different letters indicate significant differences between these two groups (*P* < 0.05).

The number and relative abundance of bacterial genera varied in the LK and QZ groups. The predominant genus was an unclassified *Enterobacteriaceae*. The relative abundance of *Nubsella*, *Acinetobacter*, *Comamonas*, *Lactococcu*s, *Xanthobacter*, *Glutamicibacter*, and *Variovorax* was higher in the QZ group than in the LK group, whereas *Serratia* showed the opposite trend (Fig 2B, S4 Table).

At the species level, the number and relative abundance of bacterial species also differed in the LK and QZ groups, ranging from 21 in the QZ group to 26 in the LK group (S5 Fig, S5 Table). Of these, the relative abundance of unclassified *Enterobacteriaceae*, *Serratia marcescens*, and *Sphingobacterium spiritivorum* was significantly higher in the LK group than in the QZ group, whereas the relative abundance of *Acinetobacter baylyi*, unclassified *Nubsella*, *Comamonas testosteroni*, *Lactococcus lactis*, *Pseudomonas geniculata*, and *Variovorax paradoxus* was significantly higher in the QZ group than in the LK group (Fig 2C, S5 Table).

In total, 84 OTUs were identified in the LK and QZ groups. Of these, 14 and 13 were identified as dominant enriched OTUs (> 0.5%) in the QZ and LK group, respectively (S6 Fig, S6 Table). The core OTUs (OTU1 and OTU2) with the highest relative abundance belonged to the Proteobacteria and unclassified *Enterobacteriaceae*. OTU3 (Proteobacteria, *Pseudomonas*) was the third most abundant in both the LK and QZ groups. The relative abundance of OTU13 (Proteobacteria, *Acinetobacter*), OTU46 (Bacteroidetes, *Nubsella*), OTU49 (Proteobacteria, *Acinetobacter*), OTU5 (Proteobacteria, *Comamonas*), OTU4 (Firmicutes, *Lactococcu*s), OTU57 (Proteobacteria, *Stenotrophomonas*), OTU66 (Proteobacteria, *Xanthobacter*), OTU29 (Actinobacteria, *Glutamicibacter*), OTU51 (Proteobacteria, *Variovorax*), and OTU48 (Bacteroidetes, *Sphingobacterium*) was higher in the QZ group than in the LK group, whereas the relative abundance of OTU44 (Bacteroidetes, *Sphingobacterium*), OTU45 (Proteobacteria, *Acinetobacter*), OTU6 (Proteobacteria, *Serratia*), and OTU76 (Bacteroidetes, *Dyadobacter*) was higher in the LK group than in the QZ group. Additionally, OTU79 was only found in the LK group (Fig 2D, S6 Table).

**Fig 3:**
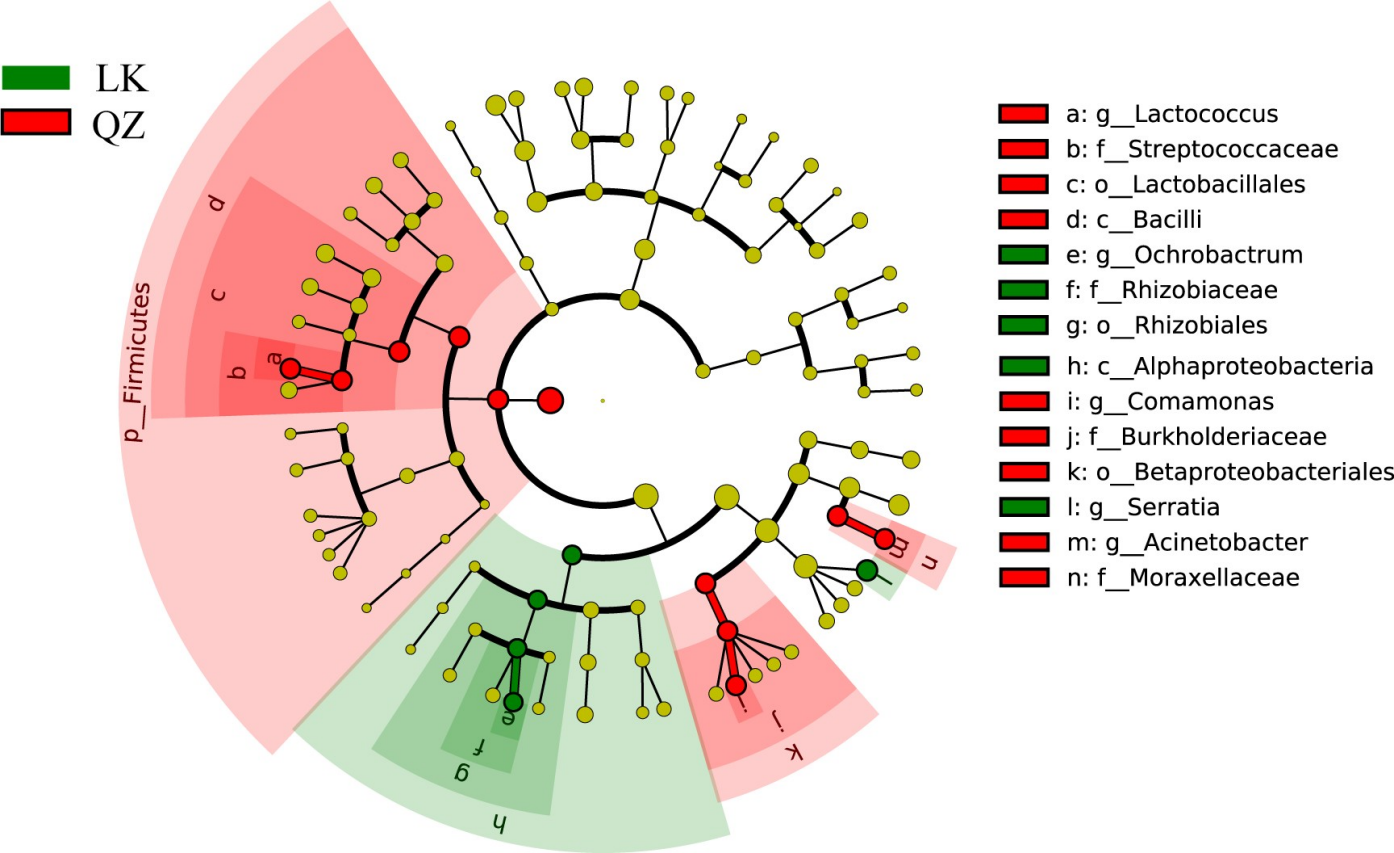
Different gut bacterial taxa abundance shown in the linear discriminant analysis effect size (LEfSe analysis) between *Solanum melongena* (QZ) and *Solanum nigrum* (LK) groups.

LEfSe analyses were performed to reveal the bacterial biomarkers of *H*. *vigintioctopunctata* that fed on two different host plants. Overall, the phylum Firmicutes was enriched in *H*. *vigintioctopunctata* that fed on the QZ group, whereas the phylum Proteobacteria was enriched in the LK group. In particular, QZ treatment enriched Bacilli of the order Lactobacillales, the family *Streptococcaceae*, and the genus *Lactococcus*; while the LK group showed more Alphaproteobacteria of the Rhizobiales order, family *Rhizobiaceae*, and genus *Ochrobactrum* (Fig 3).

**Fig 4:**
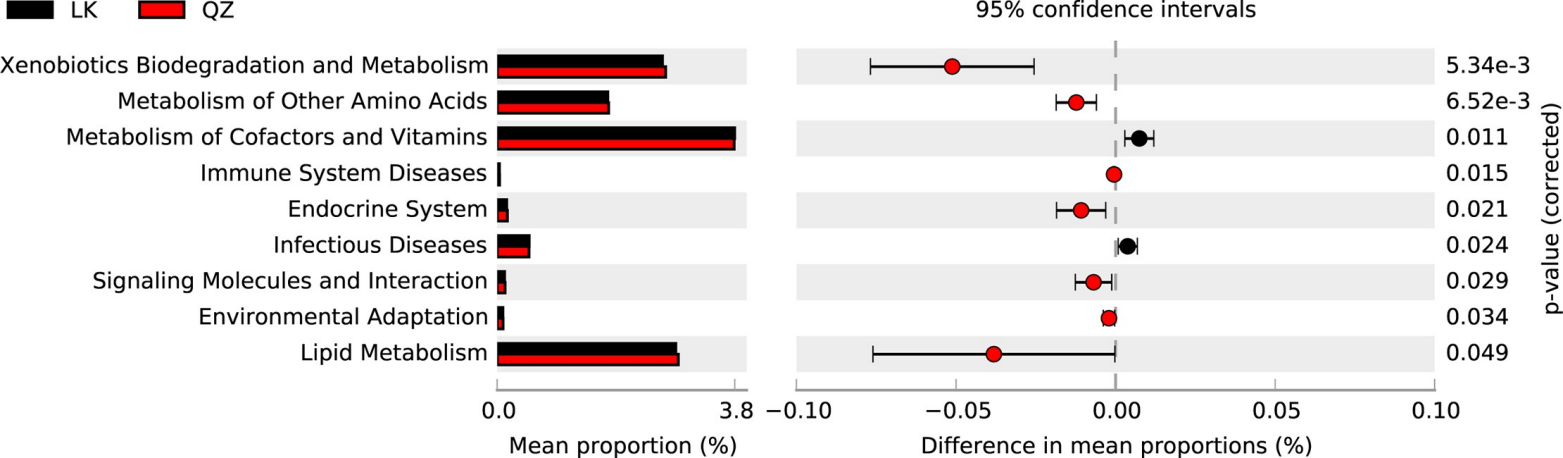
The relative abundance of predicted gut bacterial functions in the *Solanum melongena* (QZ) and *Solanum nigrum* (LK) groups, as predicted by PICRUSt using KEGG Orthologs.

The PICRUSt analysis was performed to investigate the link between gut bacteria and host metabolic changes. The significantly different functional predictions are shown in Fig 4. Specifically, pathways such as xenobiotics biodegradation and metabolism, metabolism of other amino acids, signaling molecules and interaction, environmental adaptation, and lipid metabolism were significantly higher in QZ group, whereas infectious diseases and metabolism of cofactors and vitamins were significant higher in the LK group (Fig 4).

**Fig 5:**
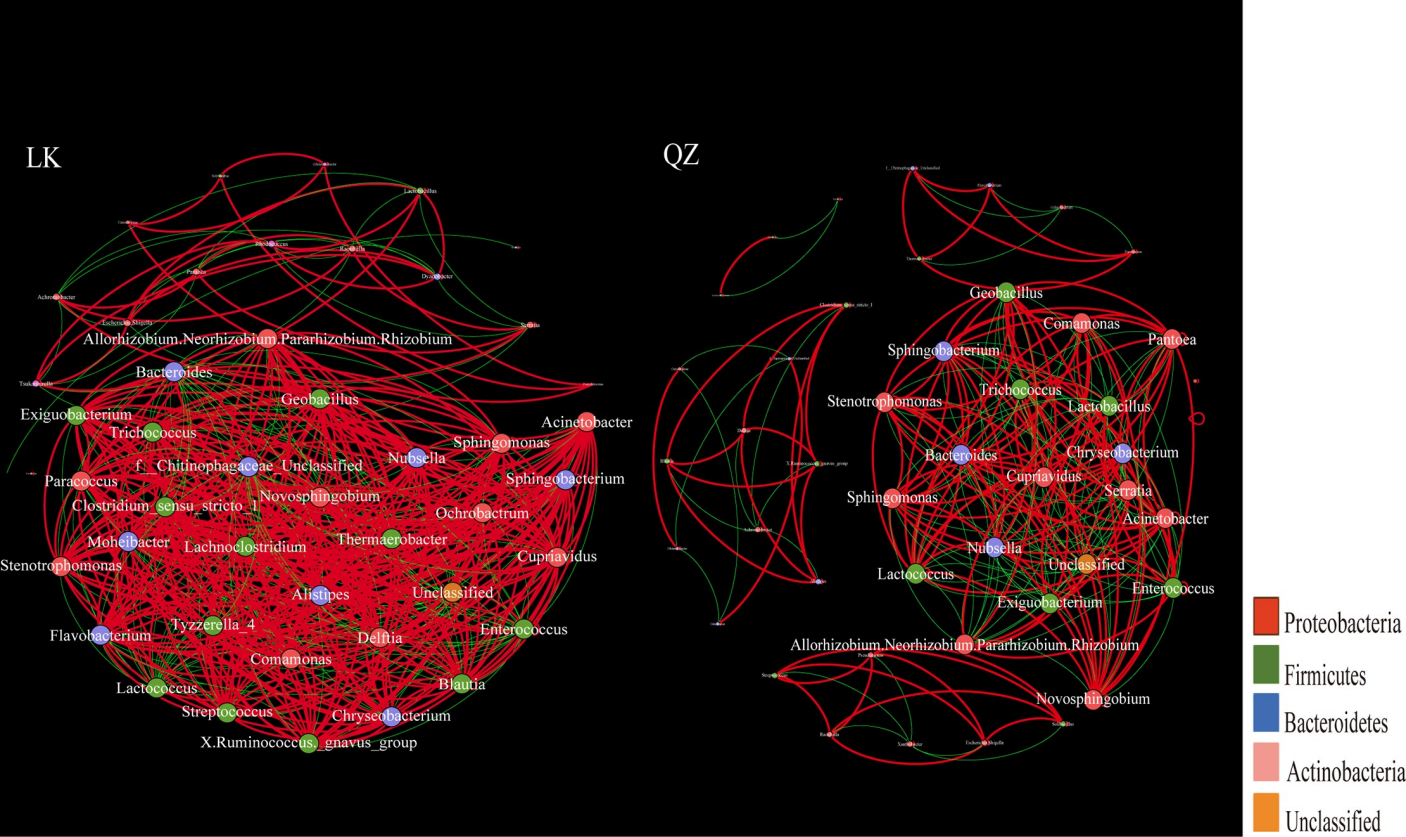
Interaction networks of gut bacteria genera from the *Solanum melongena* (QZ) and *Solanum nigrum* (LK) groups, based on correlation analysis. For each panel, the node represented unique genera, and the size of each node is proportional to the relative abundance. A red edge indicates a positive interaction between two individual nodes, while a blue edge indicates a negative interaction.

Two association networks were constructed to determine the patterns of gut bacterial communities of *H*. *vigintioctopunctata* fed with *S*. *nigrum* and *S*. *melongena*. The positive and negative correlation edges, graph density, average degree, and average weighted degree of the network in the LK group were larger than those in the QZ group, while the modularity showed an opposite trend (Fig 5, Table 3).

**Table 3:** Topological properties of the gut bacterial networks obtained from the *Solanum melongena* (QZ) and *Solanum nigrum* (LK) groups.

Network metrics	LK	QZ
Number of nodes	46	44
Number of edges	554	283
Number of positive correlations	403	172
Number of negative correlations	151	111
Average path length (APL)	1	1
Graph density	0.54	0.30
Network diameter	1	1
Average clustering coefficient (*avgCC*)	0.99	0.98
Average degree (*avgK*)	24.09	12.87
Average weighting degree	19.91	3.55
Modularity (M)	0.110	1.77

## Discussion

In the present study, we analyzed the composition and relative abundance of the gut bacterial communities of the fourth instar *H*. *vigintioctopunctata*, this is the first study to characterize the gut bacteria of *H*. *vigintioctopunctata*; our results showed that the bacterial communities were considerably influenced by feeding on the two tested host plants. It has been proposed that the main factors that influence the formation of insect gut bacterial communities are life stage, diet, and environmental factors [9, 22–24]. Our study consistent with previous studies which showed that the die can affect the bacterial community structure in many insect species [25–27], our study provides another convincible evidence that diet can influence the insect gut bacterial communities. Gut bacteria can affect the response of insects to plant defenses, and *vice versa* [28]. Here, our results revealed the presence of a high bacterial diversity, with four phyla (Proteobacteria, Bacteroidetes, Firmicutes, and Actinobacteria) present in all the *H*. *vigintioctopunctata* gut samples. Proteobacteria and *Enterobacteriaceae* were the predominant phylum and family, respectively, in both the LK and QZ groups. This is consistent with the discoveries of other scientists who reported that bacteria of the phylum Proteobacteria and family Enterobacteriaceae were the most common one in *Plutella xylostella*, *Rhynchophorus ferrugineus*, *Bactrocera dorsalis*, and *Bactrocera tau* [15, 29–31].

Our study showed that the relative abundance of Firmicutes was significantly higher in the QZ group compared to the LK group. Firmicutes have been shown to participate in energy absorption, and may influence the development of obesity and diabetes in insects, humans and mice [32,33,34]. Many studies in insects and other animals have shown that increases in the Firmicutes are related to an increased ability to harvest energy from the diet [34]. For example, *Clostridia* species belong to Firmicutes such as *C*. *thermocellum* and *C*. *ljungdahlii* are known to have a great ability to degrade the cellulose and hemicellulose, and to metabolize the amino acids [35]. Maybe the contents of cellulose and hemicellulose in the QZ leaves are higher than in the LK leaves, in other words, *H*. *vigintioctopunctata* need more Firmicutes bacteria to digestive the QZ leaves (Fig 1). In this study, the OTU79 (phylum Cyanobacteria) with a low proportion of 0.04% was only found in the LK group. The latest study divided the Cyanobacteria phylum into three classes: *Oxyphotobacteria*, “*Melainabacteria*”, and “*Sericytochromatia*”, with the latter two lacking the photosynthetic machinery [36]. One recent study showed that “*Melainabacteria*” was found from the termite gut [37], however, *Oxyphotobacteria* was detected in our study. Theoretically, the Cyanobacteria from the aphotic environment of insect gut should lose the photosynthetic capability. Therefore, our result expands the current knowledge on this Cyanobacteria group in the gut of insects; the role of Cyanobacteria in the *H*. *vigintioctopunctata* gut deserves further investigation.

The information with respect to the host-related variability in bacterial communities is very important for an integrated understanding of insect gut ecology [38]. Microorganisms in the gut can be used to enhance the resistance to transmission of pathogens, thereby protecting insects [39]. In this study, the higher relative abundance of some bacterial genera in *H*. *vigintioctopunctata* fed with *S*. *nigrum* may improve the disease resistance of this pest. One such example is *Serratia*, which has been considered a biological control agent against several plant pathogenic fungi because of the ability of its members to produce chitinase, a hydrolytic enzyme that can degrade the cell walls of fungi [40]. *S*. *marcescens* has also been reported to be a pathogen in several insects [41]. The *S*. *marcescens* strain SEN showed promise as a biological control agent of *Spodoptera litura* [42]. The impact of *Serratia* on the growth and development of *H*. *vigintioctopunctata* remains to be determined.

A previous study has shown that *S*. *nigrum* leaves have high crude protein, total carbohydrate content, and vitamin C [43]. As an antioxidant, previous studies have shown that vitamin C could reduce the microbicidal reactive oxygen species (ROS) in the gut of insects. For example, experiments with *B*. *dorsalis* showed that ingestion of a high dose of vitamin C decreased ROS levels in a dose-dependent manner, increasing the bacterial load [44]. Interestingly, the mean proportions of metabolic cofactors and vitamins were higher in the LK group which fed on *S*. *nigrum*, compared to the QZ group (Fig 4). This could help in regulating the gut bacterial community homeostasis of *H*. *vigintioctopunctata*. Therefore, further studies are needed to compare the nutrient composition in *S*. *nigrum* and *S*. *melongena*, to enhance our knowledge regarding their impact on the bacterial composition and diversity of *H*. *vigintioctopunctata*.

Different host plants had considerable impact on the bacterial networks. The greater number of edges, number of positive and negative correlations, and average degree (*avgK*) within the LK group implied that the network for LK group was complex and exhibited much more cooperation and exchange events among the dominant bacterial genera (Table 3). Moreover, the higher modularity of bacterial network in the LK group indicated that there is relatively higher system resistance to changes compared to networks in the QZ group [45]. Together, our results indicate that compared with *S*. *melongena*, *S*. *nigrum* is a better host plant to strengthen the gut bacterial network complexity and system resistance to change.

This study provides novel information regarding the bacterial diversity of *H*. *vigintioctopunctata*, demonstrating that the bacterial communities of larvae that fed on *S*. *nigrum* were different from those of larvae that fed on *S*. *melongena*. Our results support the following hypotheses: 1) different host plants have different influences on the diversity of bacterial communities associated with the *H*. *vigintioctopunctata* larvae gut; 2) the bacterial communities are dominated by a few taxa; and 3) an unclassified genus is dominant in the gut of *H*. *vigintioctopunctata*. We believe that our study makes a significant contribution to the literature because our findings advance the understanding of the bacterial community associated with the gut of an important pest, *H*. *vigintioctopunctata*.

## Supporting information

S1 Fig Rarefaction analysis of the gut bacteria in the LK and QZ groups. The numbers 1–3 in the legend represent the three biological replicate for each group.
(TIFF)

S2 Fig Hierarchical clustering of all gut bacterial samples (LK and QZ groups) based on taxon distribution. The numbers 1–3 in the legend represent the three biological replicate for each group.
(TIFF)

S3 Fig Principal coordinate analysis performed on the entire gut bacterial community in the QZ (A) and LK (B) groups.
(TIFF)

S4 Fig The relative abundance of gut bacterial family in the LK and QZ groups. (TIFF)

S5 Fig Heatmap and clustering based on bacterial species composition and abundance of the gut bacteria in the LK and QZ groups. (TIF)

S6 Fig Heatmap and clustering based on the operational taxonomic units (OTUs) in the LK and QZ groups. (TIF)

S1 Table The relative abundance of gut bacteria at the phylum level in the *Henosepilachna vigintioctopunctata*. (DOCX)

S2 Table The relative abundance of gut bacteria at the class level in the *Henosepilachna vigintioctopunctata*. (DOCX)

S3 Table The relative abundance of gut bacteria at the family level in the *Henosepilachna vigintioctopunctata*. (DOCX)

S4 Table The relative abundance of gut bacteria at the genus level in the *Henosepilachna vigintioctopunctata*. (DOCX)

S5 Table The relative abundance of bacteria at the species level in the *Henosepilachna vigintioctopunctata* gut. (DOCX)

S6 Table The relative abundance of gut bacteria at the OTU level in the *Henosepilachna vigintioctopunctata*. (DOCX)
