# Supplementary figures and images for: Host plants influence the composition of the gut bacteria in Henosepilachna vigintioctopunctata
Source: PLoS One. 2019 Oct 18;14(10):e0224213. doi: 10.1371/journal.pone.0224213 (PMC6799920; doi:10.1371/journal.pone.0224213)

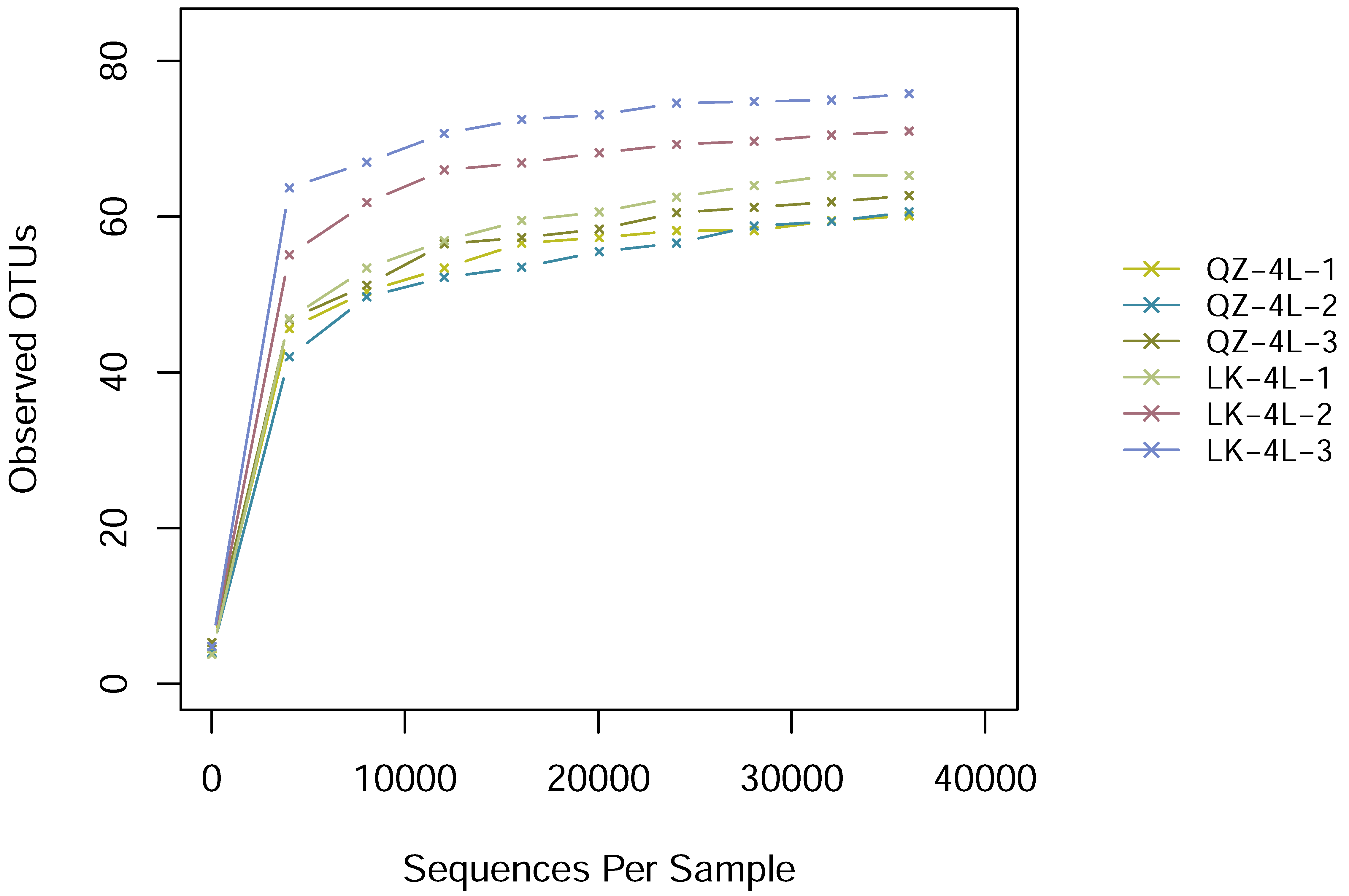

Supplement: S1 Fig — The numbers 1–3 in the legend represent the three biological replicate for each group. (TIFF) [file pone.0224213.s001.tiff]

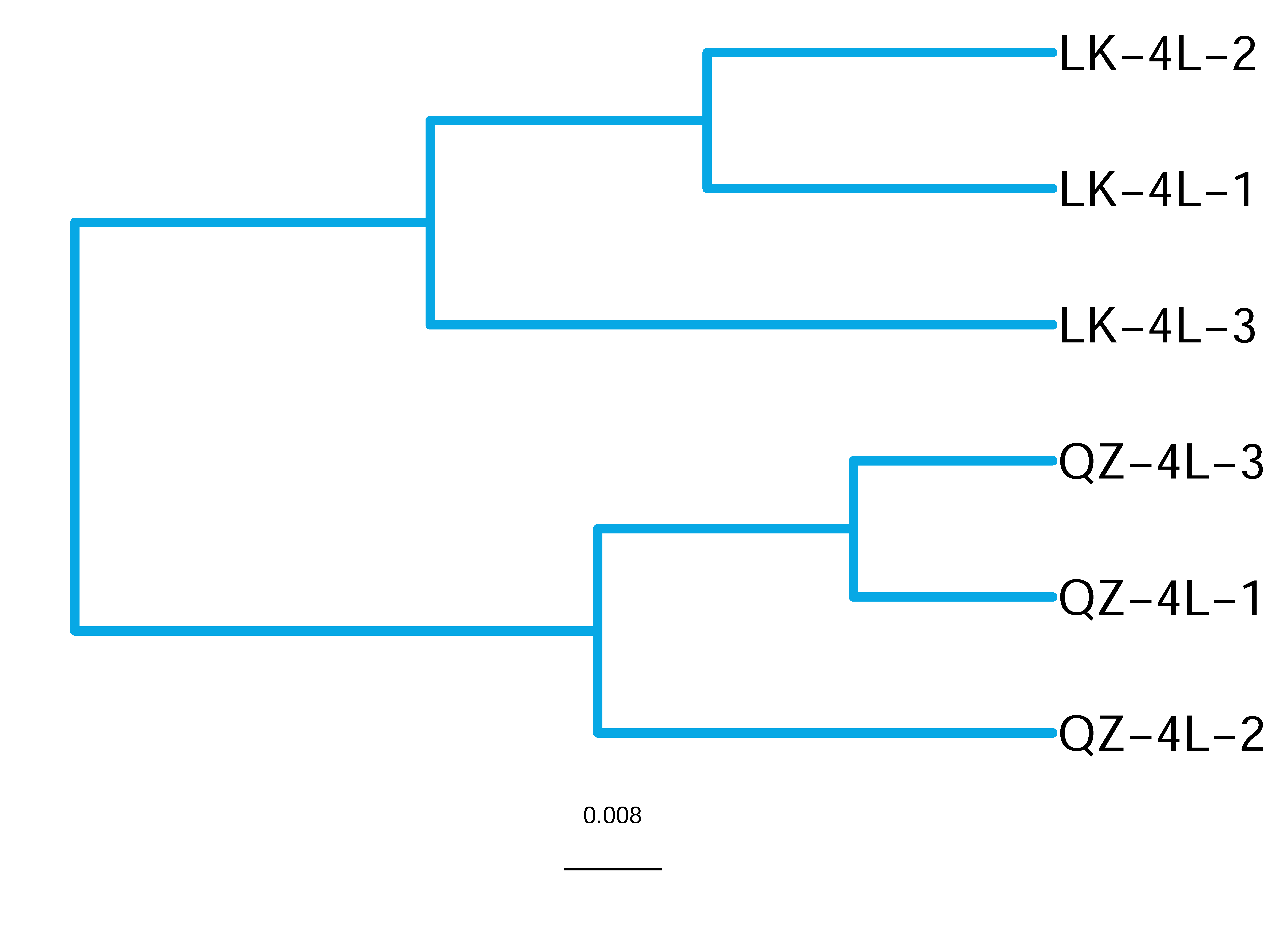

Supplement: S2 Fig — The numbers 1–3 in the legend represent the three biological replicate for each group. (TIFF) [file pone.0224213.s002.tiff]

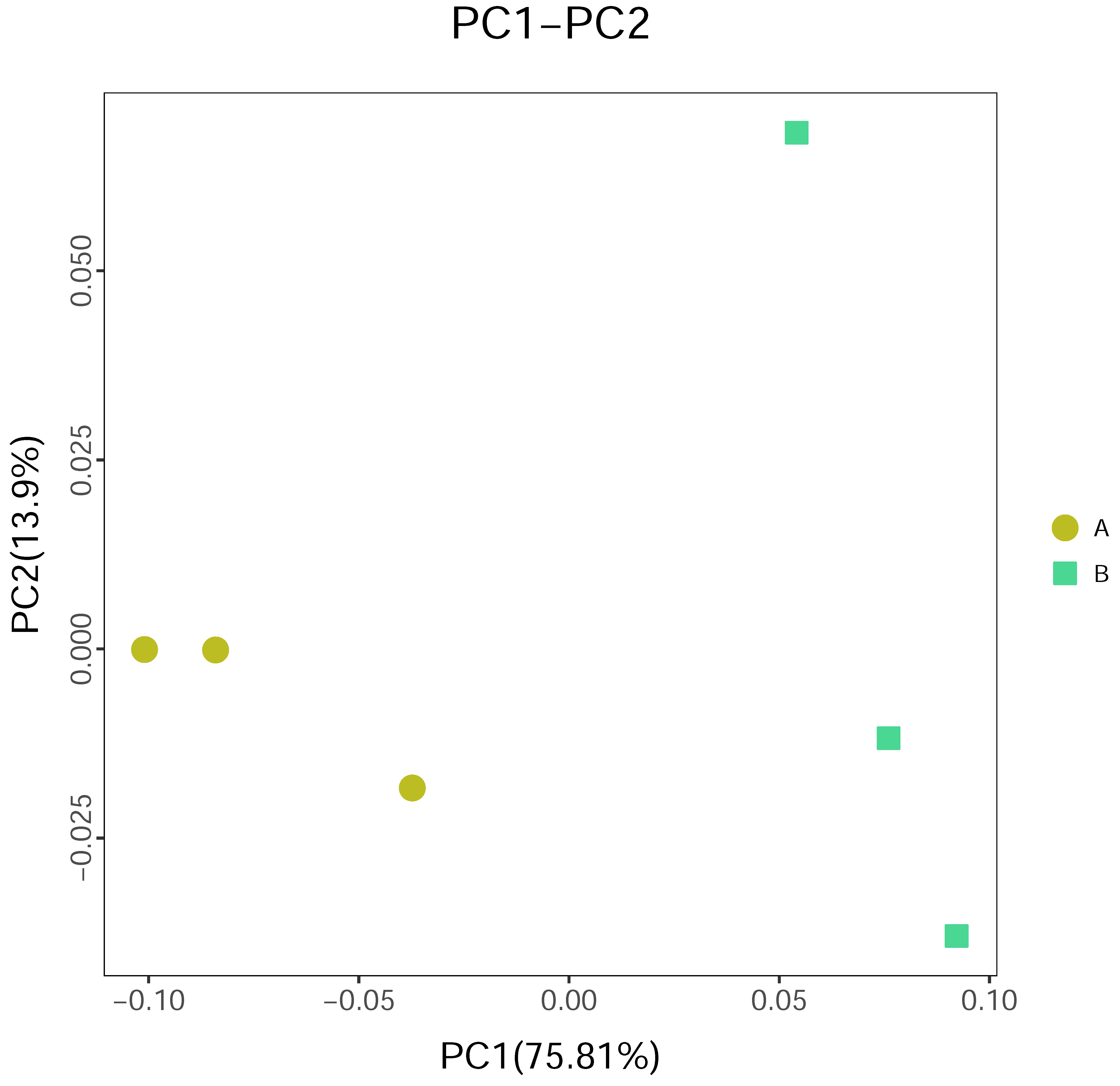

Supplement: S3 Fig — Principal coordinate analysis performed on the entire gut bacterial community in the QZ (A) and LK (B) groups. (TIFF) [file pone.0224213.s003.tiff]

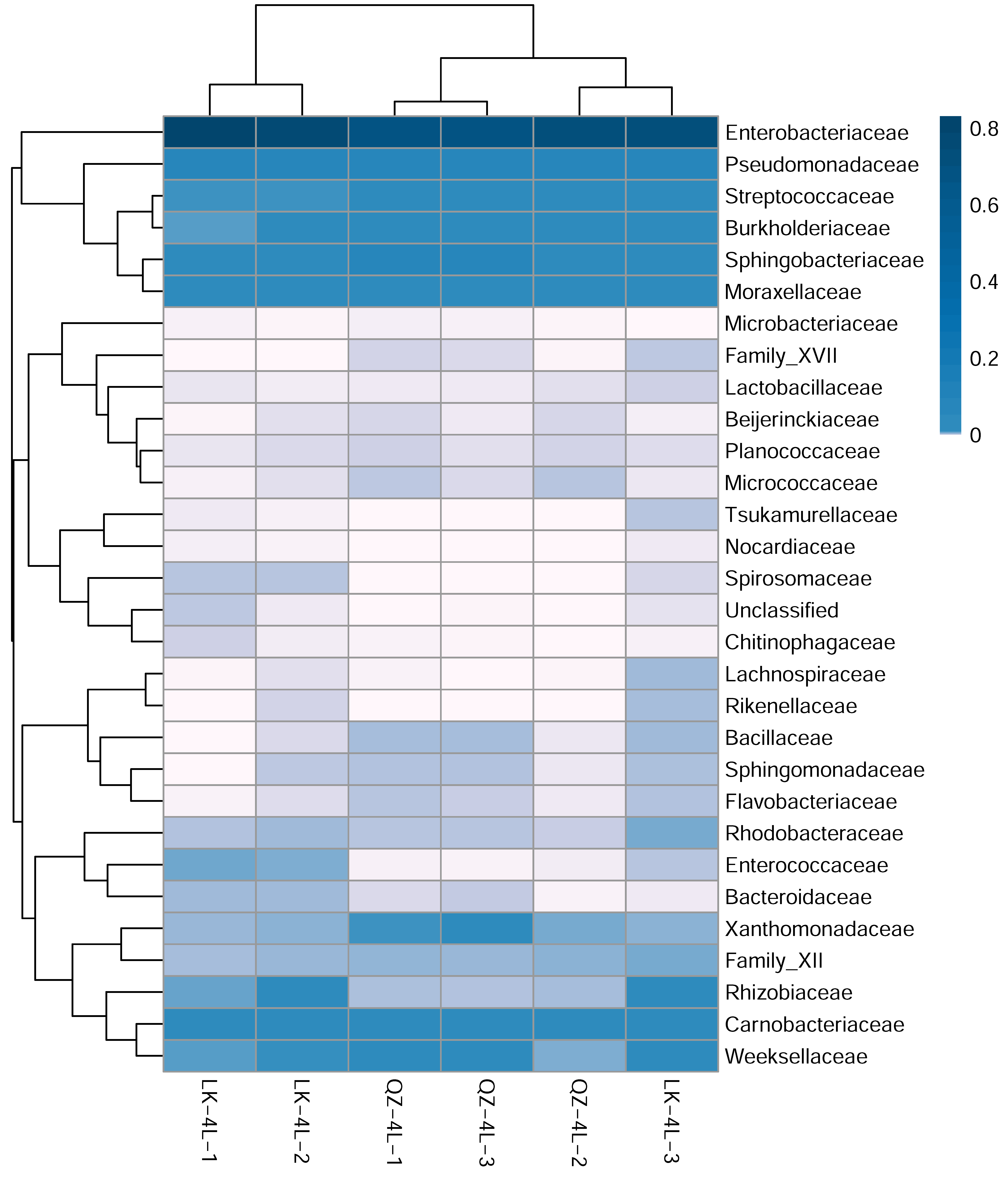

Supplement: S4 Fig — (TIFF) [file pone.0224213.s004.tiff]

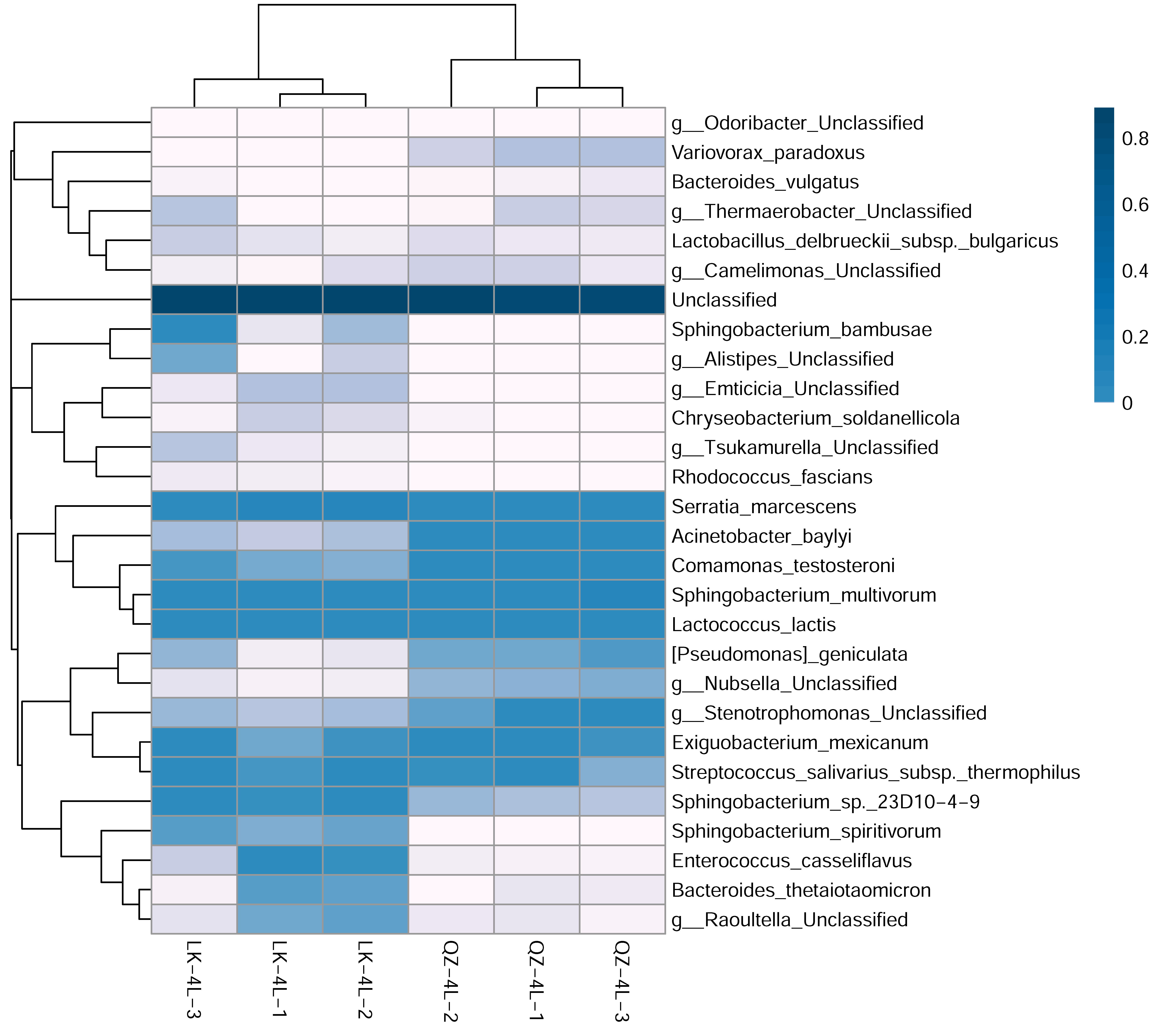

Supplement: S5 Fig — (TIF) [file pone.0224213.s005.tif]

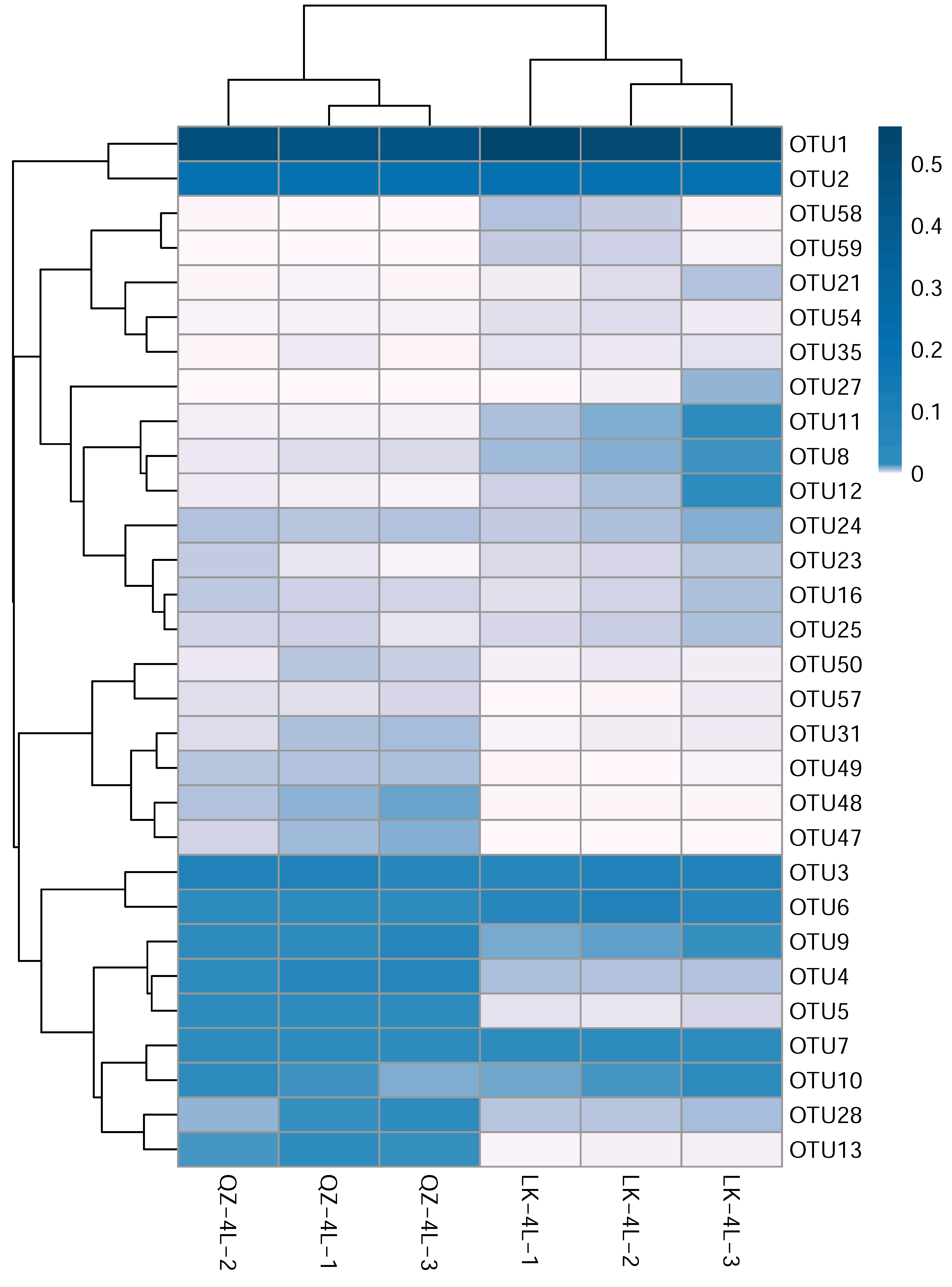

Supplement: S6 Fig — (TIF) [file pone.0224213.s006.tif]
