# Supplementary material for: Host plants influence the composition of the gut bacteria in Henosepilachna vigintioctopunctata
Source: PLoS One. 2019 Oct 18;14(10):e0224213. doi: 10.1371/journal.pone.0224213 (PMC6799920; doi:10.1371/journal.pone.0224213)
Supplement: S1 Table — (DOCX) [file pone.0224213.s007.docx]

**S1 Table. The relative abundance of gut bacteria at the phylum level in the *Henosepilachna vigintioctopunctata*.**

| Phylum | LK group (%) | QZ group  (%) | *P* |
| --- | --- | --- | --- |
| Proteobacteria | 91.74±2.48 | 86.63±2.58 | 0.07 |
| Bacteroidetes | 4.71±1.7 | 6.93±2.21 | 0.24 |
| Firmicutes | 3.43±0.77 | 6.37±0.56 | 0.01 |
| Actinobacteria | 0.08±0.03 | 0.07±0.02 | 0.62 |
| Cyanobacteria | 0.04±0.02 | 0±0 | 0.04 |
