# Supplementary material for: Host plants influence the composition of the gut bacteria in Henosepilachna vigintioctopunctata
Source: PLoS One. 2019 Oct 18;14(10):e0224213. doi: 10.1371/journal.pone.0224213 (PMC6799920; doi:10.1371/journal.pone.0224213)
Supplement: S2 Table — (DOCX) [file pone.0224213.s008.docx]

**S2 Table. The relative abundance of gut bacteria at the class level in the *Henosepilachna vigintioctopunctata*.**

| Class | LK group (%) | QZ group (%) | *P* | Phylum |
| --- | --- | --- | --- | --- |
| Gammaproteobacteria | 4.71±1.7 | 6.93±2.21 | 0.239 | Proteobacteria |
| Bacteroidia | 3.29±0.57 | 6.33±0.54 | 0.239 | Bacteroidetes |
| Bacilli | 1.83±1.31 | 0.39±0.03 | 0.002 | Firmicutes |
| Alphaproteobacteria | 0.14±0.2 | 0.04±0.03 | 0.128 | Proteobacteria |
| Clostridia | 0.08±0.03 | 0.07±0.02 | 0.445 | Firmicutes |
| Actinobacteria | 0.04±0.02 | 0±0 | 0.623 | Actinobacteria |
| Oxyphotobacteria | 0±0 | 0±0 | 0.037 | Cyanobacteria |
