# Supplementary material for: Host plants influence the composition of the gut bacteria in Henosepilachna vigintioctopunctata
Source: PLoS One. 2019 Oct 18;14(10):e0224213. doi: 10.1371/journal.pone.0224213 (PMC6799920; doi:10.1371/journal.pone.0224213)
Supplement: S3 Table — (DOCX) [file pone.0224213.s009.docx]

**S3 Table. The relative abundance of gut bacteria at the family level in the *Henosepilachna vigintioctopunctata*.**

| Family | LK group (%) | QZ group (%) | *P* | Phylum | Class | Order |
| --- | --- | --- | --- | --- | --- | --- |
| Enterobacteriaceae | 78.45±4.69 | 69.22±3.23 | 0.048 | Proteobacteria | Gammaproteobacteria | Enterobacteriales |
| Pseudomonadaceae | 7.53±1.1 | 8.04±0.83 | 0.559 | Proteobacteria | Gammaproteobacteria | Pseudomonadales |
| Sphingobacteriaceae | 3.11±1.13 | 5.96±1.82 | 0.0826 | Bacteroidetes | Bacteroidia | Sphingobacteriales |
| Moraxellaceae | 2.51±0.4 | 4.37±0.36 | 0.004 | Proteobacteria | Gammaproteobacteria | Pseudomonadales |
| Streptococcaceae | 0.94±0.11 | 4.38±0.63 | 0.001 | Firmicutes | Bacilli | Lactobacillales |
| Burkholderiaceae | 1.04±0.34 | 3.81±0.61 | 0.002 | Proteobacteria | Gammaproteobacteria | Betaproteobacteriales |
| Carnobacteriaceae | 1.41±0.41 | 1.32±0.33 | 0.780 | Firmicutes | Bacilli | Lactobacillales |
| Weeksellaceae | 1.15±0.55 | 0.88±0.34 | 0.513 | Bacteroidetes | Bacteroidia | Flavobacteriales |
| Rhizobiaceae | 1.41±1.03 | 0.17±0.04 | 0.106 | Proteobacteria | Alphaproteobacteria | Rhizobiales |
| Xanthomonadaceae | 0.38±0.06 | 0.82±0.22 | 0.029 | Proteobacteria | Gammaproteobacteria | Xanthomonadales |
| Family_XII | 0.37±0.16 | 0.37±0.05 | 0.973 | Firmicutes | Bacilli | Bacillales |
| Enterococcaceae | 0.4±0.26 | 0.01±0.01 | 0.063 | Firmicutes | Bacilli | Lactobacillales |
| Rhodobacteraceae | 0.32±0.2 | 0.06±0.01 | 0.087 | Proteobacteria | Alphaproteobacteria | Rhodobacterales |
| Bacillaceae | 0.1±0.13 | 0.17±0.13 | 0.548 | Firmicutes | Bacilli | Bacillales |
| Bacteroidaceae | 0.19±0.15 | 0.04±0.03 | 0.149 | Bacteroidetes | Bacteroidia | Bacteroidales |
| Sphingomonadaceae | 0.07±0.08 | 0.09±0.06 | 0.791 | Proteobacteria | Alphaproteobacteria | Sphingomonadales |
| Lachnospiraceae | 0.11±0.16 | 0±0.01 | 0.324 | Firmicutes | Clostridia | Clostridiales |
| Flavobacteriaceae | 0.06±0.07 | 0.05±0.03 | 0.711 | Bacteroidetes | Bacteroidia | Flavobacteriales |
| Rikenellaceae | 0.1±0.13 | 0±0 | 0.256 | Bacteroidetes | Bacteroidia | Bacteroidales |
| Planococcaceae | 0.04±0.01 | 0.04±0.01 | 0.421 | Firmicutes | Bacilli | Bacillales |
| Micrococcaceae | 0.02±0.01 | 0.05±0.01 | 0.019 | Actinobacteria | Actinobacteria | Micrococcales |
| Spirosomaceae | 0.07±0.03 | 0±0 | 0.011 | Bacteroidetes | Bacteroidia | Cytophagales |
| Lactobacillaceae | 0.03±0.02 | 0.02±0.01 | 0.348 | Firmicutes | Bacilli | Lactobacillales |
| Beijerinckiaceae | 0.01±0.02 | 0.03±0.01 | 0.144 | Proteobacteria | Alphaproteobacteria | Rhizobiales |
| Family_XVII | 0.02±0.03 | 0.03±0.03 | 0.711 | Firmicutes | Clostridia | Clostridiales |
| Tsukamurellaceae | 0.03±0.03 | 0±0 | 0.121 | Actinobacteria | Actinobacteria | Corynebacteriales |
| Chitinophagaceae | 0.03±0.02 | 0±0.01 | 0.134 | Bacteroidetes | Bacteroidia | Chitinophagales |
| Microbacteriaceae | 0±0.01 | 0.01±0.01 | 0.518 | Actinobacteria | Actinobacteria | Micrococcales |
| Nocardiaceae | 0.01±0.01 | 0±0 | 0.016 | Actinobacteria | Actinobacteria | Corynebacteriales |
| Xanthobacteraceae | 0±0 | 0.01±0.01 | 0.016 | Proteobacteria | Alphaproteobacteria | Rhizobiales |
| Caulobacteraceae | 0±0 | 0.01±0.01 | 0.116 | Proteobacteria | Alphaproteobacteria | Caulobacterales |
| Clostridiaceae_1 | 0.01±0.01 | 0±0 | 0.373 | Firmicutes | Clostridia | Clostridiales |
