# Supplementary material for: Host plants influence the composition of the gut bacteria in Henosepilachna vigintioctopunctata
Source: PLoS One. 2019 Oct 18;14(10):e0224213. doi: 10.1371/journal.pone.0224213 (PMC6799920; doi:10.1371/journal.pone.0224213)
Supplement: S4 Table — (DOCX) [file pone.0224213.s010.docx]

**S4 Table. The relative abundance of gut bacteria at the genus level in the *Henosepilachna vigintioctopunctata*.**

| Genus | LK group  (%) | QZ group (%) | *P* | Phylum | Class | Order | Family |
| --- | --- | --- | --- | --- | --- | --- | --- |
| *Unclassified* | 71.82±3.68 | 67.08±2.98 | 0.158 | Unclassified | Unclassified | Unclassified | Unclassified |
| *Pseudomonas* | 7.53±1.1 | 8.04±0.83 | 0.556 | Proteobacteria | Gammaproteobacteria | Pseudomonadales | Pseudomonadaceae |
| *Sphingobacterium* | 3.09±1.12 | 5.78±1.8 | 0.093 | Bacteroidetes | Bacteroidia | Sphingobacteriales | Sphingobacteriaceae |
| *Serratia* | 6.23±1.37 | 1.93±0.37 | 0.006 | Proteobacteria | Gammaproteobacteria | Enterobacteriales | Enterobacteriaceae |
| *Acinetobacter* | 2.51±0.4 | 4.37±0.36 | 0.004 | Proteobacteria | Gammaproteobacteria | Pseudomonadales | Moraxellaceae |
| *Lactococcus* | 0.53±0.03 | 4.09±0.67 | 0.001 | Firmicutes | Bacilli | Lactobacillales | Streptococcaceae |
| *Comamonas* | 0.86±0.3 | 3.63±0.55 | 0.002 | Proteobacteria | Gammaproteobacteria | Betaproteobacteriales | Burkholderiaceae |
| *Trichococcus* | 1.41±0.41 | 1.32±0.33 | 0.778 | Firmicutes | Bacilli | Lactobacillales | Carnobacteriaceae |
| *Chryseobacterium* | 1.01±0.34 | 0.88±0.34 | 0.678 | Bacteroidetes | Bacteroidia | Flavobacteriales | Weeksellaceae |
| *Ochrobactrum* | 1.28±0.93 | 0.09±0.02 | 0.090 | Proteobacteria | Alphaproteobacteria | Rhizobiales | Rhizobiaceae |
| *Stenotrophomonas* | 0.38±0.06 | 0.82±0.22 | 0.030 | Proteobacteria | Gammaproteobacteria | Xanthomonadales | Xanthomonadaceae |
| *Exiguobacterium* | 0.37±0.16 | 0.37±0.05 | 0.987 | Firmicutes | Bacilli | Bacillales | Family_XII |
| *Streptococcus* | 0.42±0.14 | 0.3±0.09 | 0.284 | Firmicutes | Bacilli | Lactobacillales | Streptococcaceae |
| *Enterococcus* | 0.4±0.26 | 0.01±0 | 0.061 | Firmicutes | Bacilli | Lactobacillales | Enterococcaceae |
| *Paracoccus* | 0.32±0.2 | 0.06±0.01 | 0.089 | Proteobacteria | Alphaproteobacteria | Rhodobacterales | Rhodobacteraceae |
| *Escherichia-Shigella* | 0.21±0.02 | 0.07±0.06 | 0.027 | Proteobacteria | Gammaproteobacteria | Enterobacteriales | Enterobacteriaceae |
| *Geobacillus* | 0.1±0.13 | 0.17±0.12 | 0.548 | Firmicutes | Bacilli | Bacillales | Bacillaceae |
| *Pantoea* | 0.1±0.02 | 0.14±0.05 | 0.383 | Proteobacteria | Gammaproteobacteria | Enterobacteriales | Enterobacteriaceae |
| *Bacteroides* | 0.19±0.15 | 0.03±0.03 | 0.147 | Bacteroidetes | Bacteroidia | Bacteroidales | Bacteroidaceae |
| *Nubsella* | 0.02±0.01 | 0.18±0.02 | <0.001 | Bacteroidetes | Bacteroidia | Sphingobacteriales | Sphingobacteriaceae |
| *Raoultella* | 0.18±0.13 | 0.02±0.01 | 0.102 | Proteobacteria | Gammaproteobacteria | Enterobacteriales | Enterobacteriaceae |
| *Allorhizobium-Neorhizobium*  *-Pararhizobium-Rhizobium* | 0.09±0.05 | 0.09±0.02 | 1.000 | Proteobacteria | Alphaproteobacteria | Rhizobiales | Rhizobiaceae |
| *Sphingomonas* | 0.06±0.07 | 0.09±0.06 | 0.605 | Proteobacteria | Alphaproteobacteria | Sphingomonadales | Sphingomonadaceae |
| *Moheibacter* | 0.14±0.22 | 0±0 | 0.316 | Bacteroidetes | Bacteroidia | Flavobacteriales | Weeksellaceae |
| *Achromobacter* | 0.1±0.04 | 0.02±0 | 0.028 | Proteobacteria | Gammaproteobacteria | Betaproteobacteriales | Burkholderiaceae |
| *Flavobacterium* | 0.06±0.07 | 0.05±0.03 | 0.824 | Bacteroidetes | Bacteroidia | Flavobacteriales | Flavobacteriaceae |
| *Alistipes* | 0.1±0.13 | 0±0 | 0.266 | Bacteroidetes | Bacteroidia | Bacteroidales | Rikenellaceae |
| *Cupriavidus* | 0.03±0.04 | 0.07±0.06 | 0.378 | Proteobacteria | Gammaproteobacteria | Betaproteobacteriales | Burkholderiaceae |
| *Solibacillus* | 0.04±0.01 | 0.04±0.01 | 0.263 | Firmicutes | Bacilli | Bacillales | Planococcaceae |
| *Glutamicibacter* | 0.02±0.01 | 0.06±0.01 | 0.030 | Actinobacteria | Actinobacteria | Micrococcales | Micrococcaceae |
| *Variovorax* | 0±0 | 0.07±0.02 | 0.007 | Proteobacteria | Gammaproteobacteria | Betaproteobacteriales | Burkholderiaceae |
| *Delftia* | 0.05±0.04 | 0.02±0 | 0.223 | Proteobacteria | Gammaproteobacteria | Betaproteobacteriales | Burkholderiaceae |
| *Emticicia* | 0.06±0.03 | 0±0 | 0.034 | Bacteroidetes | Bacteroidia | Cytophagales | Spirosomaceae |
| *Lactobacillus* | 0.03±0.02 | 0.03±0.01 | 0.614 | Firmicutes | Bacilli | Lactobacillales | Lactobacillaceae |
| *Camelimonas* | 0.02±0.02 | 0.04±0.01 | 0.173 | Proteobacteria | Alphaproteobacteria | Rhizobiales | Beijerinckiaceae |
| *Thermaerobacter* | 0.02±0.03 | 0.03±0.02 | 0.693 | Firmicutes | Clostridia | Clostridiales | Family_XVII |
| *Ruminococcus_gnavus_group* | 0.05±0.07 | 0±0 | 0.331 | Firmicutes | Clostridia | Clostridiales | Lachnospiraceae |
| *Blautia* | 0.03±0.05 | 0±0 | 0.316 | Firmicutes | Clostridia | Clostridiales | Lachnospiraceae |
| *Tsukamurella* | 0.03±0.03 | 0±0 | 0.100 | Actinobacteria | Actinobacteria | Corynebacteriales | Tsukamurellaceae |
| *Chitinophagaceae_Unclassified* | 0.03±0.02 | 0±0 | 0.133 | Bacteroidetes | Bacteroidia | Chitinophagales | Chitinophagaceae |
| *Novosphingobium* | 0.02±0.01 | 0.01±0.01 | 0.316 | Proteobacteria | Alphaproteobacteria | Sphingomonadales | Sphingomonadaceae |
| *Tyzzerella_4* | 0.02±0.02 | 0±0 | 0.196 | Firmicutes | Clostridia | Clostridiales | Lachnospiraceae |
| *Dyadobacter* | 0.01±0.01 | 0±0 | 0.033 | Bacteroidetes | Bacteroidia | Cytophagales | Spirosomaceae |
| *Lachnoclostridium* | 0.01±0.02 | 0±0 | 0.350 | Firmicutes | Clostridia | Clostridiales | Lachnospiraceae |
| *Rhodococcus* | 0.01±0.01 | 0±0 | 0.018 | Actinobacteria | Actinobacteria | Corynebacteriales | Nocardiaceae |
| *Xanthobacter* | 0±0 | 0.01±0 | 0.004 | Proteobacteria | Alphaproteobacteria | Rhizobiales | Xanthobacteraceae |
| *Clostridium_sensu_stricto_1* | 0.01±0.01 | 0±0 | 0.275 | Firmicutes | Clostridia | Clostridiales | Clostridiaceae_1 |
| *Veillonella* | 0±0 | 0±0 | 0.374 | Firmicutes | Negativicutes | Selenomonadales | Veillonellaceae |
| *Saprospiraceae_Unclassified* | 0±0 | 0±0 | 0.374 | Bacteroidetes | Bacteroidia | Chitinophagales | Saprospiraceae |
| *Odoribacter* | 0±0 | 0±0 | 0.374 | Bacteroidetes | Bacteroidia | Bacteroidales | Marinifilaceae |
