# Supplementary material for: Host plants influence the composition of the gut bacteria in Henosepilachna vigintioctopunctata
Source: PLoS One. 2019 Oct 18;14(10):e0224213. doi: 10.1371/journal.pone.0224213 (PMC6799920; doi:10.1371/journal.pone.0224213)
Supplement: S5 Table — (DOCX) [file pone.0224213.s011.docx]

**S5 Table. The relative abundance of gut bacteria at the species level in the *Henosepilachna vigintioctopunctata*.**

| Species | LK group (%) | QZ group (%) | *P* | Phylum | Class | Order | Family | Genus |
| --- | --- | --- | --- | --- | --- | --- | --- | --- |
| *Unclassified* | 88.69±0.73 | 83.44±2.22 | 0.018 | Unclassified | Unclassified | Unclassified | Unclassified | *Unclassified* |
| *Serratia_marcescens* | 6.1±1.44 | 1.92±0.37 | 0.008 | Proteobacteria | Gammaproteobacteria | Enterobacteriales | Enterobacteriaceae | *Serratia* |
| *Sphingobacterium_multivorum* | 1.23±0.2 | 4.09±1.32 | 0.020 | Bacteroidetes | Bacteroidia | Sphingobacteriales | Sphingobacteriaceae | *Sphingobacterium* |
| *Lactococcus_lactis* | 0.53±0.03 | 4.09±0.67 | 0.001 | Firmicutes | Bacilli | Lactobacillales | Streptococcaceae | *Lactococcus* |
| *Comamonas_testosteroni* | 0.24±0.06 | 3.11±0.53 | 0.001 | Proteobacteria | Gammaproteobacteria | Betaproteobacteriales | Burkholderiaceae | *Comamonas* |
| *Acinetobacter_baylyi* | 0.09±0.03 | 1.42±0.09 | <0.001 | Proteobacteria | Gammaproteobacteria | Pseudomonadales | Moraxellaceae | *Acinetobacter* |
| *Sphingobacterium_sp._23D10-4-9* | 0.8±0.62 | 0.1±0.04 | 0.122 | Bacteroidetes | Bacteroidia | Sphingobacteriales | Sphingobacteriaceae | *Sphingobacterium* |
| *Exiguobacterium_mexicanum* | 0.37±0.16 | 0.37±0.05 | 0.987 | Firmicutes | Bacilli | Bacillales | Family_XII | *Exiguobacterium* |
| *Streptococcus_salivarius* | 0.42±0.14 | 0.3±0.09 | 0.284 | Firmicutes | Bacilli | Lactobacillales | Streptococcaceae | *Streptococcus* |
| *Stenotrophomonas_Unclassified* | 0.1±0.04 | 0.48±0.19 | 0.025 | Proteobacteria | Gammaproteobacteria | Xanthomonadales | Xanthomonadaceae | *Streptococcus* |
| *[Pseudomonas]_geniculata* | 0.06±0.08 | 0.26±0.04 | 0.017 | Proteobacteria | Gammaproteobacteria | Xanthomonadales | Xanthomonadaceae | *Stenotrophomonas* |
| *Enterococcus_casseliflavus* | 0.27±0.19 | 0.01±0.01 | 0.082 | Firmicutes | Bacilli | Lactobacillales | Enterococcaceae | *Enterococcus* |
| *Sphingobacterium_spiritivorum* | 0.25±0.04 | 0±0 | <0.001 | Bacteroidetes | Bacteroidia | Sphingobacteriales | Sphingobacteriaceae | *Sphingobacterium* |
| *Bacteroides_thetaiotaomicron* | 0.19±0.15 | 0.01±0.01 | 0.125 | Bacteroidetes | Bacteroidia | Bacteroidales | Bacteroidaceae | *Bacteroides* |
| *Nubsella_Unclassified* | 0.02±0.01 | 0.18±0.02 | <0.001 | Bacteroidetes | Bacteroidia | Sphingobacteriales | Sphingobacteriaceae | *Nubsella* |
| *Raoultella_Unclassified* | 0.18±0.13 | 0.02±0.01 | 0.102 | Proteobacteria | Gammaproteobacteria | Enterobacteriales | Enterobacteriaceae | *Raoultella* |
| *Sphingobacterium_bambusae* | 0.18±0.18 | 0±0 | 0.161 | Bacteroidetes | Bacteroidia | Sphingobacteriales | Sphingobacteriaceae | *Sphingobacterium* |
| *Alistipes_Unclassified* | 0.1±0.13 | 0±0 | 0.266 | Bacteroidetes | Bacteroidia | Bacteroidales | Rikenellaceae | *Alistipes* |
| *Variovorax_paradoxus* | 0±0 | 0.07±0.02 | 0.007 | Proteobacteria | Gammaproteobacteria | Betaproteobacteriales | Burkholderiaceae | *Variovorax* |
| *Emticicia_Unclassified* | 0.06±0.03 | 0±0 | 0.034 | Bacteroidetes | Bacteroidia | Cytophagales | Spirosomaceae | *Emticicia* |
| *Lactobacillus_delbrueckii* | 0.03±0.02 | 0.03±0.01 | 0.614 | Firmicutes | Bacilli | Lactobacillales | Lactobacillaceae | *Lactobacillus* |
| *Camelimonas_Unclassified* | 0.02±0.02 | 0.04±0.01 | 0.173 | Proteobacteria | Alphaproteobacteria | Rhizobiales | Beijerinckiaceae | *Camelimonas* |
| *Thermaerobacter_Unclassified* | 0.02±0.03 | 0.03±0.02 | 0.693 | Firmicutes | Clostridia | Clostridiales | Family_XVII | *Thermaerobacter* |
| *Chryseobacterium_soldanellicola* | 0.03±0.02 | 0±0 | 0.088 | Bacteroidetes | Bacteroidia | Flavobacteriales | Weeksellaceae | *Chryseobacterium* |
| *Tsukamurella_Unclassified* | 0.03±0.03 | 0±0 | 0.100 | Actinobacteria | Actinobacteria | Corynebacteriales | Tsukamurellaceae | *Tsukamurella* |
| *Bacteroides_vulgatus* | 0±0 | 0.01±0.01 | 0.180 | Bacteroidetes | Bacteroidia | Bacteroidales | Bacteroidaceae | *Bacteroides* |
| *Rhodococcus_fascians* | 0.01±0.01 | 0±0 | 0.018 | Actinobacteria | Actinobacteria | Corynebacteriales | Nocardiaceae | *Rhodococcus* |
| *Odoribacter_Unclassified* | 0±0 | 0±0 | 0.374 | Bacteroidetes | Bacteroidia | Bacteroidales | Marinifilaceae | *Odoribacter* |
