# Supplementary material for: Host plants influence the composition of the gut bacteria in Henosepilachna vigintioctopunctata
Source: PLoS One. 2019 Oct 18;14(10):e0224213. doi: 10.1371/journal.pone.0224213 (PMC6799920; doi:10.1371/journal.pone.0224213)
Supplement: S6 Table — (DOCX) [file pone.0224213.s012.docx]

**S6 Table. The relative abundance of gut bacteria at the OTU level in the *Henosepilachna vigintioctopunctata*.**

| OTU | LK group (%) | QZ group (%) | *P* | Phylum | Class | Order | Family | Genus | Species |
| --- | --- | --- | --- | --- | --- | --- | --- | --- | --- |
| OTU01 | 51.24±3.55 | 46.86±2.12 | 0.140 | Proteobacteria | Gammaproteobacteria | Enterobacteriales | Enterobacteriaceae | *norank* | *norank* |
| OTU02 | 19.89±0.53 | 20.18±0.86 | 0.647 | Proteobacteria | Gammaproteobacteria | Enterobacteriales | Enterobacteriaceae | *norank* | *norank* |
| OTU03 | 7.52±1.1 | 8.03±0.83 | 0.554 | Proteobacteria | Gammaproteobacteria | Pseudomonadales | Pseudomonadaceae | *Pseudomonas* | *norank* |
| OTU04 | 0.53±0.03 | 4.09±0.67 | 0.001 | Firmicutes | Bacilli | Lactobacillales | Streptococcaceae | *Lactococcus* | *Lactococcus_lactis* |
| OTU05 | 0.24±0.06 | 3.11±0.53 | 0.001 | Proteobacteria | Gammaproteobacteria | Betaproteobacteriales | Burkholderiaceae | *Comamonas* | *Comamonas_testosteroni* |
| OTU06 | 6.1±1.44 | 1.92±0.37 | 0.008 | Proteobacteria | Gammaproteobacteria | Enterobacteriales | Enterobacteriaceae | *Serratia* | *Serratia_marcescens* |
| OTU07 | 1.85±0.17 | 2.19±0.39 | 0.240 | Proteobacteria | Gammaproteobacteria | Pseudomonadales | Moraxellaceae | *Acinetobacter* | *norank* |
| OTU08 | 0.98±0.36 | 0.24±0.06 | 0.025 | Bacteroidetes | Bacteroidia | Flavobacteriales | Weeksellaceae | *Chryseobacterium* | *norank* |
| OTU09 | 1.19±0.2 | 3.28±1.02 | 0.025 | Bacteroidetes | Bacteroidia | Sphingobacteriales | Sphingobacteriaceae | *Sphingobacterium* | *Sphingobacterium_multivorum* |
| OTU10 | 1.41±0.41 | 1.32±0.33 | 0.778 | Firmicutes | Bacilli | Lactobacillales | Carnobacteriaceae | *Trichococcus* | *norank* |
| OTU11 | 1.28±0.93 | 0.09±0.02 | 0.090 | Proteobacteria | Alphaproteobacteria | Rhizobiales | Rhizobiaceae | *Ochrobactrum* | *norank* |
| OTU12 | 0.8±0.62 | 0.1±0.04 | 0.122 | Bacteroidetes | Bacteroidia | Sphingobacteriales | Sphingobacteriaceae | *Sphingobacterium* | *Sphingobacterium_sp._23D10-4-9* |
| OTU13 | 0.09±0.03 | 1.42±0.09 | <0.001 | Proteobacteria | Gammaproteobacteria | Pseudomonadales | Moraxellaceae | *Acinetobacter* | *Acinetobacter_baylyi* |
| OTU14 | 0.09±0.05 | 0.09±0.02 | 1.000 | Proteobacteria | Alphaproteobacteria | Rhizobiales | Rhizobiaceae | *Allorhizobium-Neorhizobium-Pararhizobium-Rhizobium* | *norank* |
| OTU15 | 0.1±0.13 | 0±0 | 0.266 | Bacteroidetes | Bacteroidia | Bacteroidales | Rikenellaceae | *Alistipes* | *uncultured_organism* |
| OTU16 | 0.37±0.16 | 0.37±0.05 | 0.987 | Firmicutes | Bacilli | Bacillales | Family_XII | *Exiguobacterium* | *Exiguobacterium_mexicanum* |
| OTU17 | 0.14±0.22 | 0±0 | 0.316 | Bacteroidetes | Bacteroidia | Flavobacteriales | Weeksellaceae | *Moheibacter* | *norank* |
| OTU18 | 0.02±0.03 | 0.03±0.02 | 0.693 | Firmicutes | Clostridia | Clostridiales | Family_XVII | *Thermaerobacter* | *Ambiguous_taxa* |
| OTU19 | 0.1±0.13 | 0.17±0.12 | 0.548 | Firmicutes | Bacilli | Bacillales | Bacillaceae | *Geobacillus* | *norank* |
| OTU20 | 0.13±0.08 | 0±0 | 0.045 | Proteobacteria | Gammaproteobacteria | Enterobacteriales | Enterobacteriaceae | *Serratia* | *norank* |
| OTU21 | 0.29±0.19 | 0.04±0 | 0.086 | Proteobacteria | Alphaproteobacteria | Rhodobacterales | Rhodobacteraceae | *Paracoccus* | *norank* |
| OTU22 | 0.06±0.07 | 0.09±0.06 | 0.605 | Proteobacteria | Alphaproteobacteria | Sphingomonadales | Sphingomonadaceae | *Sphingomonas* | *norank* |
| OTU23 | 0.36±0.1 | 0.22±0.18 | 0.305 | Proteobacteria | Gammaproteobacteria | Pseudomonadales | Moraxellaceae | *Acinetobacter* | *norank* |
| OTU24 | 0.62±0.25 | 0.49±0.04 | 0.427 | Proteobacteria | Gammaproteobacteria | Betaproteobacteriales | Burkholderiaceae | *Comamonas* | *norank* |
| OTU25 | 0.42±0.14 | 0.3±0.09 | 0.284 | Firmicutes | Bacilli | Lactobacillales | Streptococcaceae | *Streptococcus* | *Streptococcus_salivarius_subsp._thermophilus* |
| OTU26 | 0.18±0.18 | 0±0 | 0.161 | Bacteroidetes | Bacteroidia | Sphingobacteriales | Sphingobacteriaceae | *Sphingobacterium* | *Sphingobacterium_bambusae* |
| OTU27 | 0.29±0.41 | 0±0 | 0.293 | Proteobacteria | Gammaproteobacteria | Enterobacteriales | Enterobacteriaceae | *norank* | *norank* |
| OTU28 | 0.52±0.1 | 1.25±0.42 | 0.043 | Bacteroidetes | Bacteroidia | Sphingobacteriales | Sphingobacteriaceae | *Sphingobacterium* | *norank* |
| OTU29 | 0.02±0.01 | 0.06±0.01 | 0.030 | Actinobacteria | Actinobacteria | Micrococcales | Micrococcaceae | *Glutamicibacter* | *norank* |
| OTU30 | 0.1±0.04 | 0.02±0 | 0.028 | Proteobacteria | Gammaproteobacteria | Betaproteobacteriales | Burkholderiaceae | *Achromobacter* | *norank* |
| OTU31 | 0.1±0.04 | 0.48±0.19 | 0.025 | Proteobacteria | Gammaproteobacteria | Xanthomonadales | Xanthomonadaceae | *Stenotrophomonas* | *uncultured_organism* |
| OTU32 | 0.04±0.01 | 0.04±0.01 | 0.263 | Firmicutes | Bacilli | Bacillales | Planococcaceae | *Solibacillus* | *norank* |
| OTU33 | 0.05±0.06 | 0±0 | 0.224 | Proteobacteria | Alphaproteobacteria | Rhizobiales | Rhizobiaceae | *norank* | *norank* |
| OTU34 | 0.05±0.07 | 0±0 | 0.331 | Firmicutes | Clostridia | Clostridiales | Lachnospiraceae | *[Ruminococcus]_gnavus_group* | *norank* |
| OTU35 | 0.21±0.02 | 0.07±0.06 | 0.027 | Proteobacteria | Gammaproteobacteria | Enterobacteriales | Enterobacteriaceae | *Escherichia-Shigella* | *norank* |
| OTU36 | 0±0 | 0±0 | 0.374 | Bacteroidetes | Bacteroidia | Cytophagales | Spirosomaceae | *Dyadobacter* | *norank* |
| OTU37 | 0.02±0.02 | 0±0 | 0.196 | Firmicutes | Clostridia | Clostridiales | Lachnospiraceae | *Tyzzerella_4* | *norank* |
| OTU38 | 0.03±0.05 | 0±0 | 0.316 | Firmicutes | Clostridia | Clostridiales | Lachnospiraceae | *Blautia* | *norank* |
| OTU39 | 0.03±0.02 | 0±0 | 0.133 | Bacteroidetes | Bacteroidia | Chitinophagales | Chitinophagaceae | *uncultured* | *norank* |
| OTU40 | 0.05±0.04 | 0.02±0 | 0.223 | Proteobacteria | Gammaproteobacteria | Betaproteobacteriales | Burkholderiaceae | *Delftia* | *norank* |
| OTU41 | 0.01±0.01 | 0±0 | 0.275 | Firmicutes | Clostridia | Clostridiales | Clostridiaceae_1 | *Clostridium_sensu_stricto_1* | *norank* |
| OTU42 | 0.03±0.04 | 0.07±0.06 | 0.378 | Proteobacteria | Gammaproteobacteria | Betaproteobacteriales | Burkholderiaceae | *Cupriavidus* | *norank* |
| OTU43 | 0.01±0.01 | 0±0 | 0.018 | Actinobacteria | Actinobacteria | Corynebacteriales | Nocardiaceae | *Rhodococcus* | *Rhodococcus_fascians* |
| OTU44 | 0.25±0.04 | 0±0 | <0.001 | Bacteroidetes | Bacteroidia | Sphingobacteriales | Sphingobacteriaceae | *Sphingobacterium* | *Sphingobacterium_spiritivorum* |
| OTU45 | 0.01±0 | 0±0 | 0.003 | Proteobacteria | Gammaproteobacteria | Pseudomonadales | Moraxellaceae | *Acinetobacter* | *norank* |
| OTU46 | 0.02±0.01 | 0.18±0.02 | <0.001 | Bacteroidetes | Bacteroidia | Sphingobacteriales | Sphingobacteriaceae | *Nubsella* | *uncultured_bacterium* |
| OTU47 | 0±0 | 0.64±0.29 | 0.019 | Bacteroidetes | Bacteroidia | Flavobacteriales | Weeksellaceae | *Chryseobacterium* | *norank* |
| OTU48 | 0.03±0.01 | 0.82±0.3 | 0.011 | Bacteroidetes | Bacteroidia | Sphingobacteriales | Sphingobacteriaceae | *Sphingobacterium* | *Sphingobacterium_multivorum* |
| OTU49 | 0.04±0.02 | 0.53±0.06 | <0.001 | Proteobacteria | Gammaproteobacteria | Pseudomonadales | Moraxellaceae | *Acinetobacter* | *norank* |
| OTU50 | 0.13±0.03 | 0.33±0.15 | 0.082 | Bacteroidetes | Bacteroidia | Sphingobacteriales | Sphingobacteriaceae | *Sphingobacterium* | *norank* |
| OTU51 | 0±0 | 0.07±0.02 | 0.007 | Proteobacteria | Gammaproteobacteria | Betaproteobacteriales | Burkholderiaceae | *Variovorax* | *Variovorax_paradoxus* |
| OTU52 | 0±0 | 0.01±0.01 | 0.346 | Proteobacteria | Alphaproteobacteria | Caulobacterales | Caulobacteraceae | *norank* | *norank* |
| OTU53 | 0.03±0.02 | 0.03±0.01 | 0.614 | Firmicutes | Bacilli | Lactobacillales | Lactobacillaceae | *Lactobacillus* | *Lactobacillus_delbrueckii_subsp._bulgaricus* |
| OTU54 | 0.22±0.07 | 0.07±0.01 | 0.028 | Proteobacteria | Gammaproteobacteria | Xanthomonadales | Xanthomonadaceae | *Stenotrophomonas* | *norank* |
| OTU55 | 0.02±0.02 | 0.04±0.01 | 0.173 | Proteobacteria | Alphaproteobacteria | Rhizobiales | Beijerinckiaceae | *Camelimonas* | *uncultured_bacterium* |
| OTU56 | 0±0 | 0±0 | 0.374 | Firmicutes | Negativicutes | Selenomonadales | Veillonellaceae | *Veillonella* | *norank* |
| OTU57 | 0.06±0.08 | 0.26±0.04 | 0.017 | Proteobacteria | Gammaproteobacteria | Xanthomonadales | Xanthomonadaceae | *Stenotrophomonas* | *[Pseudomonas]_geniculata* |
| OTU58 | 0.32±0.24 | 0.02±0.01 | 0.104 | Proteobacteria | Gammaproteobacteria | Enterobacteriales | Enterobacteriaceae | *norank* | *norank* |
| OTU59 | 0.27±0.19 | 0.01±0.01 | 0.082 | Firmicutes | Bacilli | Lactobacillales | Enterococcaceae | *Enterococcus* | *Enterococcus_casseliflavus* |
| OTU60 | 0.03±0.01 | 0.02±0.01 | 0.286 | Proteobacteria | Alphaproteobacteria | Rhodobacterales | Rhodobacteraceae | *Paracoccus* | *norank* |
| OTU61 | 0±0 | 0±0 | 0.374 | Bacteroidetes | Bacteroidia | Bacteroidales | Marinifilaceae | *Odoribacter* | *Ambiguous_taxa* |
| OTU62 | 0±0 | 0±0 | 0.374 | Bacteroidetes | Bacteroidia | Chitinophagales | Saprospiraceae | *uncultured* | *norank* |
| OTU63 | 0.1±0.02 | 0.14±0.05 | 0.383 | Proteobacteria | Gammaproteobacteria | Enterobacteriales | Enterobacteriaceae | *Pantoea* | *norank* |
| OTU64 | 0±0 | 0.02±0.01 | 0.041 | Proteobacteria | Gammaproteobacteria | Betaproteobacteriales | Burkholderiaceae | *Comamonas* | *norank* |
| OTU65 | 0.06±0.07 | 0.05±0.03 | 0.824 | Bacteroidetes | Bacteroidia | Flavobacteriales | Flavobacteriaceae | *Flavobacterium* | *norank* |
| OTU66 | 0±0 | 0.01±0 | 0.004 | Proteobacteria | Alphaproteobacteria | Rhizobiales | Xanthobacteraceae | *Xanthobacter* | *norank* |
| OTU67 | 0±0 | 0±0 | 0.374 | Proteobacteria | Alphaproteobacteria | norank | norank | *norank* | *norank* |
| OTU68 | 0±0 | 0±0 | 0.643 | Proteobacteria | Gammaproteobacteria | Pseudomonadales | Moraxellaceae | *Acinetobacter* | *norank* |
| OTU69 | 0±0 | 0.01±0.01 | 0.180 | Bacteroidetes | Bacteroidia | Bacteroidales | Bacteroidaceae | *Bacteroides* | *Bacteroides_vulgatus* |
| OTU70 | 0±0 | 0.01±0.01 | 0.163 | Bacteroidetes | Bacteroidia | Bacteroidales | Bacteroidaceae | *Bacteroides* | *norank* |
| OTU71 | 0.19±0.15 | 0.01±0.01 | 0.125 | Bacteroidetes | Bacteroidia | Bacteroidales | Bacteroidaceae | *Bacteroides* | *Bacteroides_thetaiotaomicron* |
| OTU72 | 0±0 | 0±0 | 1.000 | Proteobacteria | Gammaproteobacteria | Enterobacteriales | Enterobacteriaceae | *Serratia* | *norank* |
| OTU73 | 0.13±0.07 | 0±0 | 0.030 | Firmicutes | Bacilli | Lactobacillales | Enterococcaceae | *Enterococcus* | *norank* |
| OTU74 | 0.03±0.02 | 0±0 | 0.088 | Bacteroidetes | Bacteroidia | Flavobacteriales | Weeksellaceae | *Chryseobacterium* | *Chryseobacterium_soldanellicola* |
| OTU75 | 0.18±0.13 | 0.02±0.01 | 0.102 | Proteobacteria | Gammaproteobacteria | Enterobacteriales | Enterobacteriaceae | *Raoultella* | *Ambiguous_taxa* |
| OTU76 | 0.01±0 | 0±0 | 0.012 | Bacteroidetes | Bacteroidia | Cytophagales | Spirosomaceae | *Dyadobacter* | *norank* |
| OTU77 | 0±0 | 0.01±0.01 | 0.234 | Actinobacteria | Actinobacteria | Micrococcales | Microbacteriaceae | *norank* | *norank* |
| OTU78 | 0.03±0.03 | 0±0 | 0.100 | Actinobacteria | Actinobacteria | Corynebacteriales | Tsukamurellaceae | *Tsukamurella* | *Ambiguous_taxa* |
| OTU79 | 0.04±0.02 | 0±0 | 0.031 | Cyanobacteria | Oxyphotobacteria | Chloroplast | norank | *norank* | *norank* |
| OTU80 | 0.06±0.03 | 0±0 | 0.034 | Bacteroidetes | Bacteroidia | Cytophagales | Spirosomaceae | *Emticicia* | *Ambiguous_taxa* |
| OTU81 | 0.01±0.01 | 0±0 | 0.252 | Proteobacteria | Gammaproteobacteria | Pseudomonadales | Pseudomonadaceae | *Pseudomonas* | *norank* |
| OTU82 | 0.01±0.02 | 0±0 | 0.350 | Firmicutes | Clostridia | Clostridiales | Lachnospiraceae | *Lachnoclostridium* | *norank* |
| OTU83 | 0.17±0.11 | 0±0 | 0.057 | Proteobacteria | Gammaproteobacteria | Pseudomonadales | Moraxellaceae | *Acinetobacter* | *norank* |
| OTU84 | 0.02±0.01 | 0.01±0.01 | 0.316 | Proteobacteria | Alphaproteobacteria | Sphingomonadales | Sphingomonadaceae | *Novosphingobium* | *norank* |
